# Language Impairments in ASD Resulting from a Failed Domestication of the Human Brain

**DOI:** 10.3389/fnins.2016.00373

**Published:** 2016-08-29

**Authors:** Antonio Benítez-Burraco, Wanda Lattanzi, Elliot Murphy

**Affiliations:** ^1^Department of Philology, University of Huelva Huelva, Spain; ^2^Institute of Anatomy and Cell Biology, Università Cattolica del Sacro Cuore Rome, Italy; ^3^Division of Psychology and Language Sciences, University College London London, UK

**Keywords:** autism, domestication, language evolution, neural oscillations, language deficits

## Abstract

Autism spectrum disorders (ASD) are pervasive neurodevelopmental disorders entailing social and cognitive deficits, including marked problems with language. Numerous genes have been associated with ASD, but it is unclear how language deficits arise from gene mutation or dysregulation. It is also unclear why ASD shows such high prevalence within human populations. Interestingly, the emergence of a modern faculty of language has been hypothesized to be linked to changes in the human brain/skull, but also to the process of self-domestication of the human species. It is our intention to show that people with ASD exhibit less marked domesticated traits at the morphological, physiological, and behavioral levels. We also discuss many ASD candidates represented among the genes known to be involved in the “domestication syndrome” (the constellation of traits exhibited by domesticated mammals, which seemingly results from the hypofunction of the neural crest) and among the set of genes involved in language function closely connected to them. Moreover, many of these genes show altered expression profiles in the brain of autists. In addition, some candidates for domestication and language-readiness show the same expression profile in people with ASD and chimps in different brain areas involved in language processing. Similarities regarding the brain oscillatory behavior of these areas can be expected too. We conclude that ASD may represent an abnormal ontogenetic itinerary for the human faculty of language resulting in part from changes in genes important for the “domestication syndrome” and, ultimately, from the normal functioning of the neural crest.

## Introduction

Autism spectrum disorders (ASD) are pervasive neurodevelopmental conditions characterized by several and severe cognitive and social deficits, including language and communication problems, repetitive and stereotypical behavior, and problems with social interaction (Bailey et al., 1996). In DSM-V, language deficits are no longer explicitly postulated as a central feature of ASD because they are subsumed in its distinctive communication problems. Nevertheless, it is clear that ASD entails a typical language profile and language developmental path (reviewed in Benítez-Burraco and Murphy, 2016; see also Tager-Flusberg et al., 2005; Tager-Flusberg, 2006; Eigsti et al., 2007; Bourguignon et al., 2012). Because of the masking effect of a variable IQ, and the variable degree of functionality exhibited by ASD patients, it is difficult to hypothesize a core language deficit in this condition. The impairment of the oromotor function has been claimed to account for expressive language problems in some autistic subjects (Belmonte et al., 2013). Comprehension problems seemingly result from other underlying deficit(s), including a reduced effect of semantic priming (Preissler, 2008), problems with phonological processing (Lindgren et al., 2009), or impairment of procedural memory (Walenski et al., 2006).

At the neural level, ASD entails atypical development, wiring and interconnection of areas involved in language processing (Stefanatos and Baron, 2011; Bourguignon et al., 2012). Not surprisingly, functional differences in language processing tasks of ASD compared with unaffected subjects have been attested as well (Courchesne and Pierce, 2005; Scott-Van Zeeland et al., 2010a,b). For instance, microstructural anomalies and reduced lateralization patterns have been observed in the arcuate fasciculus of ASD patients (Fletcher et al., 2010), suggesting that a constraint on the integrative processes during development may contribute to language impairment in this condition (Schipul et al., 2011). We also wish to highlight both increased and decreased intra- and inter-hemispheric connectivity (Hahamy et al., 2015), and abnormal responses to linguistic stimuli (reviewed in Stefanatos and Baron, 2011, pp 259–262). Intriguingly, the ASD phenotype is characterized by increased intrinsic functional connectivity during the first years of life (the time window where language is acquired) and reduced connectivity in adolescent and adult states (Uddin et al., 2013).

In spite of this growing body of neurobiological data, a comprehensive view of language processing in the ASD brain is still lacking. Specifically, ASD studies need to move beyond simplistic models of language processing and focus instead on how collections of brain areas jointly engaged in specific, impaired cognitive operations (see Fedorenko and Thompson-Schill, 2014, for a general discussion). This is a real challenge, provided that abnormal brain profiles are not expected to easily map on to anomalous categories or computations of linguistic theories (see Poeppel, 2012; Murphy, 2016a, for discussion). We have recently proposed a translational theory of language deficits in ASD as amounting to abnormal patterns of brain rhythms (Benítez-Burraco and Murphy, 2016); although a clarification and empirical validation of this hypothesis is still pending.

Finally, we wish to emphasize that ASD has been associated with sequence variants, copy number variation (CNVs), and/or changes in the expression patterns of an extensive number of genes (Geschwind and State, 2015). Despite the remarkable genetic heterogeneity, it is noteworthy that all these genes tend to converge on specific pathways and neural mechanisms, functionally relevant in this condition and expected to account for its associated deficits (Willsey and State, 2015). Specifically, several candidates for language impairment in ASD have been proposed, including *MET, CTTNBP2, EN2, NBEA, HRAS*, and *PTEN* (Comings et al., 1996; Naqvi et al., 2000; Cheung et al., 2001; Castermans et al., 2003; Benayed et al., 2005; Campbell et al., 2006). Nonetheless, the gap between genes and language deficits in ASD still remains open (see Jeste and Geschwind, 2014, for a general discussion, and Benítez-Burraco and Murphy, 2016, for a specific discussion on candidates for language dysfunction in ASD).

The aim of this paper is to contribute to the bridging of the gap between the genetic backdrop and language deficits observed in ASD. To this end, we will primarily focus on language evolution. There exists a strong, deep link between evolution and (abnormal) development. Recently-evolved neural networks seem to be more sensitive to damage because of their lower levels of resilience (Toro et al., 2010). As a consequence, aspects of brain development and function that are preferably impaired in modern populations are expected to be involved in recently evolved, human-specific cognitive abilities. Some comprehensive accounts of the human condition set against the cognitive profiles of other primates have been recently put forth (Seed and Tomasello, 2010; Platt et al., 2016). Comparative genomics also provides valuable information about the sources of the observed differences and similarities in the human genome (Rogers and Gibbs, 2014; Franchini and Pollard, 2015). Likewise, we are beginning to achieve an advanced understanding of the genetic changes that occurred after our split from extinct hominins (Pääbo, 2014; Zhou et al., 2015). We expect that the same factors that prompted the transition from an ape-like cognition to our specific mode of cognition are involved in the etiology of cognitive disorders involving language deficits and, particularly, of ASD (see Benítez-Burraco, 2016a, for a general discussion).

In what follows, the focus is placed on one aspect of this evolutionary process: the self-domestication of the human species. At present, we have a decent understanding of how our language-readiness (that is, our species-specific ability to learn and use language) may have evolved. Accordingly, among the changes brought about by human evolution, one very relevant aspect is the ability to transcend (better than other species) the signature limits of core knowledge systems and thus go beyond modular boundaries (Mithen, 1996; Spelke, 2003; Carruthers, 2006; Hauser, 2009; Boeckx, 2011; Wynn and Coolidge, 2011). As hypothesized in Boeckx and Benítez-Burraco (2014a), our language-readiness boils down to this enhanced cognitive ability, but also to its embedding inside cognitive systems responsible for interpretation (*thought*) and externalization (*speech*). This language-readiness was seemingly brought about by specific changes in the skull/brain developmental path (resulting in a more globular brain), which entailed new patterns of long-distance connections among distributed neurons and, ultimately, new patterns of brain rhythmicity, including an adequate degree and pattern of cortical inhibition. Interestingly, brain rhythms are heritable components of brain function (Linkenkaer-Hansen et al., 2007; Hall et al., 2011) and have been linked to computational primitives of language (Murphy, 2015a,b, 2016a), allowing for a good explanation (and not just a description) of linguistic computation (and of language deficits) at the brain level, and specifically, for a satisfactory mapping of language deficits to neural dysfunction and its genetic basis in ASD (Benítez-Burraco and Murphy, 2016). We have found many candidates for ASD among the genes known to be involved in the emergence of language-readiness (Benítez-Burraco and Boeckx, 2015).

At the same time, the emergence of modern-like languages (and perhaps of core aspects of language too) was seemingly favored by changes in the cultural niche of our ancestors. The archeological record shows that cognitive modernity (encompassing language-readiness) did not automatically entail behavioral modernity (seemingly resulting from using fully-fledged languages), which only appeared long after the emergence of anatomically-modern humans (AMHs) together with changes in human cultural dynamics. Current linguistic research has shown that aspects of linguistic complexity (including core aspects of grammatical knowledge) correlate with aspects of social complexity (Wray and Grace, 2007; Lupyan and Dale, 2010). Moreover, core properties of human languages (like duality of patterning) can develop in response to environmental pressure, as research into emergent sign languages has nicely illustrated, implying that they cannot be regarded as part of the biological endowment (see Benítez-Burraco, 2016b, for discussion). Importantly, language acquisition by the child demands a prolonged socialization window that enables her to receive the proper amount of triggering stimuli and to interact with other conspecifics. All this means that the intrinsic cognitive machinery may be not enough for granting the acquisition of a successful tool for linguistic cognition and that the environment has to be of the right kind too (see Sterelny, 2011 on behavioral modernity set against cognitive modernity).

It has been hypothesized that the social conditions (or the *cultural niche*) that facilitated the enhancement of linguistic structure through a cultural mechanism were brought about by a process of human self-domestication (see Thomas, 2014, for details, and Hare and Tomasello, 2005; Deacon, 2009, on relaxed selective pressures resulting from self-domestication as explanations of the emergence of key aspects of behavioral modernity). Different factors may have contributed to human self-domestication, from adaptation to the human-made environment to selection against aggression to sexual selection. We have hypothesized (Benítez-Burraco et al., in press) that the very changes that brought about our globular skull/brain and our language-readiness may have also fuelled the emergence of a (self-domesticated) phenotype in the human species. Accordingly, we have found numerous links between the candidates for globularization and language-readiness, and genes important for the development and function of neural crest cells (NCC). Indeed, the hypofunction of the neural crest (NC) has been claimed to account for the constellation of distinctive traits observed in domestic mammals (the “domestication syndrome”) (Wilkins et al., 2014).

Because of the deep link between evolution and development, we expect that examining the signatures of the domesticated phenotype in people with ASD contributes to a better understanding of etiology of ASD, and specifically, of language deficits in this condition. In a recent paper Reser (2014) found similarities between autism and species of solitary mammals. Although the focus was put on behavior, the author suggests that future research will benefit from investigating the neurobiological, genetic and epigenetic causes of these similarities. Here we try to push research in this direction. The paper is structured as follows. First, we provide a general account of the domesticated traits that are absent or attenuated in ASD. Then we move to the genes and focus on candidates for ASD that are found among the set of genes involved in the domestication syndrome and the evolution of language-readiness, as characterized in Benítez-Burraco et al. (in press), showing that they exhibit a distinctive expression profile in the brain of autists. Finally, we compare the ASD phenotype with wild primates, focusing on the expression profile of these genes, but also on oscillatory signatures of areas important for language processing, considering that language impairment in ASD can be interpreted as an “oscillopathic” condition (see Benítez-Burraco and Murphy, 2016). We will conclude that ASD (and language deficits in ASD) can be viewed as an abnormal ontogenetic itinerary for the human faculty of language, resulting in part from changes in genes important for the domestication syndrome and seemingly from changes in the normal functioning of the NC.

## Domestic traits in the ASD phenotype

**Figure 1:**
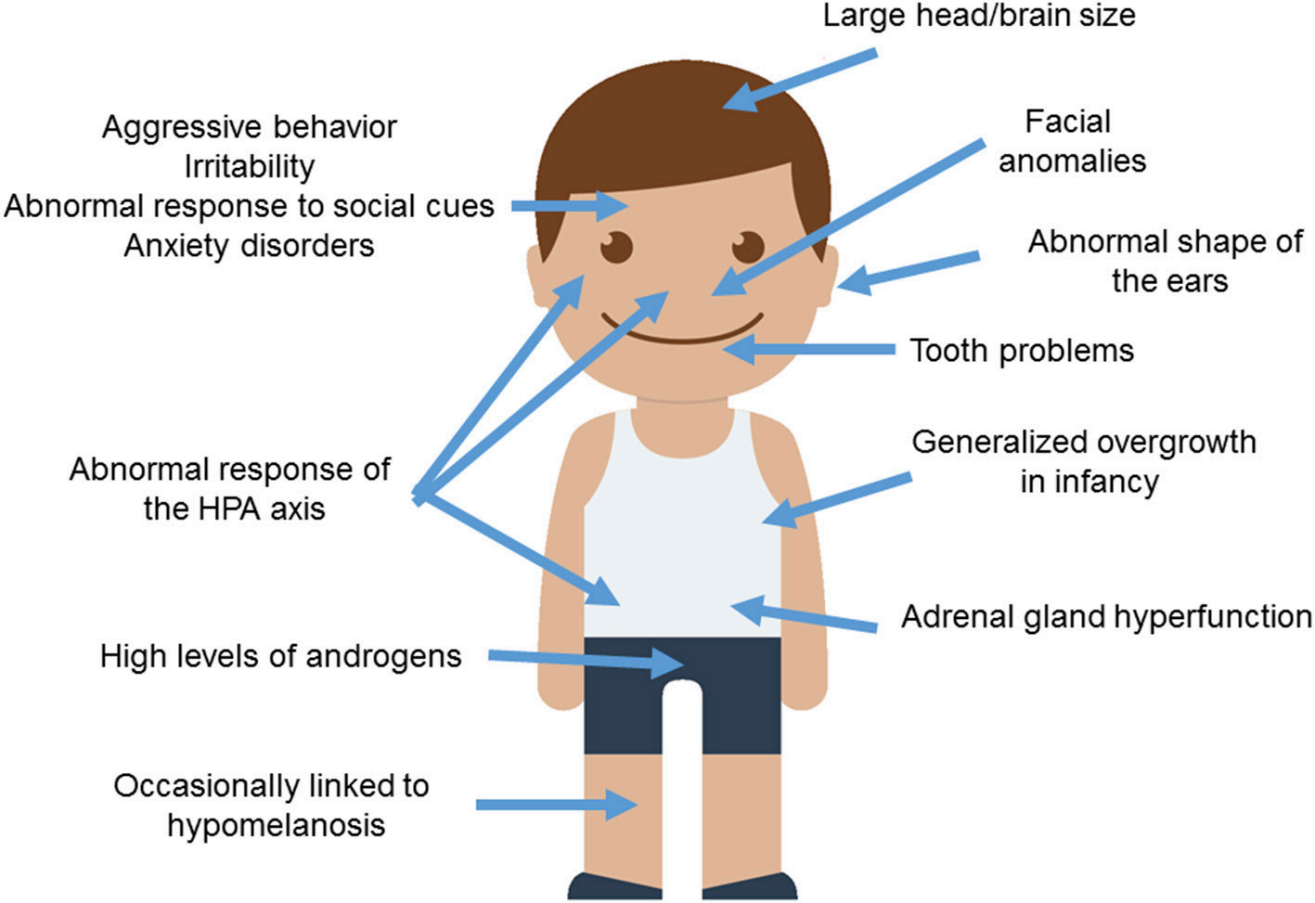
**Anomalous presentation of domesticated traits in people with ASD**. Main clinical features observed in ASD patients and concerning the domestication syndrome are shown. The child diagram was gathered from Iconfinder output (available at http://www.iconfinder.com/icons/525448/boy_child_kid_male_man_person_white_icon).

Wilkins et al. (2014) provide a comprehensive summary of traits known to be modified in domesticated mammals, many of them concerning the cranial region. These include changes in ear size and shape, changes in the orofacial area (including shorter snouts and smaller jaws), changes in dentition (particularly, smaller teeth), and a reduced brain capacity (specifically, of components of the forebrain such as the amygdala or parts of the limbic system). Other distinctive traits commonly found in domesticated strains are depigmentation, neoteny, shorter reproductive cycles, and increased docility, which is thought to result from adrenal size reduction and adrenal hypofunction as well as from reduced levels of stress hormones (including adrenocorticoids, adrenocorticotropic hormone, cortisol, and corticosterone). This delayed adrenal maturation also involves a hypofunction of the sympathetic nervous system and an increase of the duration of the immaturity of the hypothalamic-pituitary-adrenal system (the HPA axis), which provides the animal with a longer socialization window. According to Wilkins et al. (2014), the multiple phenotypic traits that characterize the domestication syndrome emerge as unselected by-products from a developmental reduction in NCC inputs, resulting from selection for tameness. Interestingly, compared to extinct hominins, AMHs exhibit a number of domesticated traits, including reduced brains (at least during the last 50,000 years), changes in dentition, reduction of aggressiveness, and retention of juvenile characteristics (see Thomas, 2014, for details). Intriguingly, most of these features are generally attenuated in ASD (Figure 1).

To begin with, ASD subjects show significant differences with healthy controls regarding minor physical anomalies, particularly in the craniofacial region (assumed to result from deviations during fetal development and suggested to constitute external markers of atypical brain growth) (Tripi et al., 2008; Manouilenko et al., 2014). Specifically, in adults the abnormal shape of the ears is robustly associated with autistic traits, with higher scores correlating with poorer functioning (Manouilenko et al., 2014). Regarding the changes in the orofacial region, prepubertal boys with ASD show significant differences in facial morphology compared to typically developing (TD) boys (Aldridge et al., 2011). This distinctive facial phenotype is more pronounced in subjects with severe symptoms, significant cognitive impairment, and language regression (Obafemi-Ajayi et al., 2015). Concerning tooth peculiarities, children with ASD show greater abnormalities in dentition, including missing teeth, diastemas, or reverse overjets (Luppanapornlarp et al., 2010). With respect to brain size, head circumference is significantly larger in people with ASD, with nearly 15% suffering from macrocephaly. Higher brain volumes correlate with lower functioning abilities; indeed nearly 9% ASD individuals exhibit brain overgrowth (Sacco et al., 2015). It is worth noticing that higher head circumference and brain size values are observed only during early childhood (Fukumoto et al., 2008; Courchesne et al., 2011, although see Raznahan et al., 2013), particularly when ASD is presented with regression (Nordahl et al., 2011). Typically, early brain overgrowth is followed by a decrease in structural volumes (Courchesne et al., 2011). Although brain overgrowth may result from a dysregulation of the overall systemic growth (see below), it is thought to impact on cognition. This is believed to occur as a result of the reduced networking efficiency among widespread regions of the cortex, due to the increased long-distance connections (Lewis et al., 2013). Specifically, people with ASD show increased volumes of the amygdala (Mosconi et al., 2009; Murphy et al., 2012), which correlate with the severity of their social and communication impairments (Schumann et al., 2009). In the TD population, higher amygdala volumes are associated with poorer language abilities in infancy (Ortiz-Mantilla et al., 2010).

Regarding the behavioral traits associated with the domestication syndrome, we wish to highlight that aggressive behaviors are frequent in children with ASD (with about 25% of them having scores in the clinical range), and correlate with lower cognitive outcomes (Hill et al., 2014). Children with ASD display more reactive than proactive aggression attitudes (Farmer et al., 2015). Likewise, irritability is also commonly observed in affected individuals (Mikita et al., 2015). Additionally, ASD is commonly found to be comorbid with generalized anxiety disorder (Hollocks et al., 2014; Bitsika et al., 2015). Several studies have been carried out to learn more about the physiological basis of this anomalous response to the social environment. Interestingly, higher serum cortisol responses are usually found in children with ASD, particularly after stressor stimulation, when prolonged duration and recovery of cortisol elevation is also observed (Spratt et al., 2012). Moreover, children with ASD show a distinctive diurnal rhythm of cortisol compared to their TD peers; this involves elevated cortisol levels at the end of the day and dampened linear decline across the day in some children (Tomarken et al., 2015). Dysregulation of the diurnal rhythm as a whole has been found in low functioning ASD (Taylor and Corbett, 2014). Also, anxiety symptoms correlate with high cortisol levels in ASD pediatric patients (Bitsika et al., 2015). Plasma levels of adrenocorticotropic hormone are also significantly higher in children with ASD, and correlate positively with the severity of the symptoms (Hamza et al., 2010). The HPA axis in ASD responds in a more sluggish way to physiological or physical manipulation. Accordingly, Taylor and Corbett (2014) found hyper-responsiveness of the HPA axis when unpleasant stimuli or relatively benign social situations are involved, whereas they observed hypo-responsiveness in conditions involving social evaluative threat. On the whole, the HPA axis may be more reactive to stress in social anxiety disorder and ASD (Spratt et al., 2012; Jacobson, 2014). Because children with autism and anxiety disorders show a blunted cortisol response to psychosocial stress, and given that reduced cortisol responsiveness is significantly related to increased anxiety symptoms, Hollocks et al. (2014) suggested that a non-adaptive physiological response to psychosocial stress may exist in ASD.

Finally, it is worth considering some other traits commonly observed in domesticated mammals: neoteny, alterations of reproductive cycles, and pigmentation changes. Regarding neotenic features, it is noteworthy that children with ASD exhibit an early generalized overgrowth (van Daalen et al., 2007; Fukumoto et al., 2008; Chawarska et al., 2011). Typically, boys with ASD show increased body size at birth and during infancy, with postnatal overgrowth correlating with lower adaptive functioning, greater severity of social deficits, and poorer verbal skills (Chawarska et al., 2011; Campbell et al., 2014). Interestingly, higher levels of androgens are found in children and adolescents with ASD. This correlates with the severity of autistic traits and might account for the precocious puberty also reported in this condition (El-Baz et al., 2014). These findings emphasize the role of elevated pre- and postnatal testosterone levels in the liability for ASD (see Hauth et al., 2014). Testosterone significantly affects brain development, particularly targeting the hypothalamus, the amygdala and the hippocampus, impacting on aspects of memory consolidation (Filová et al., 2013). High perinatal testosterone concentration negatively correlates with early vocabulary development in TD boys (Hollier et al., 2013). Interestingly, children with elevated androgen levels due to congenital adrenal hyperplasia show atypical patterns of brain asymmetry in the perisylvian areas, and language/learning disabilities (Plante et al., 1996). Less data on reproductive functions in females is available, due to the lower prevalence of ASD among women. Nevertheless, women with ASD reported significantly more irregular menstrual cycles and dysmenorrhea (Ingudomnukul et al., 2007; Hamilton et al., 2011). Likewise, an increase in premenstrual syndrome has been observed in women with ASD (Obaydi and Puri, 2008; Hamilton et al., 2011), who are more likely to exhibit behavioral issues related to the onset of periods (Burke et al., 2009). In addition, delayed age of menarche seems to correlate with the severity of autistic traits (Hergüner and Hergüner, 2016). These findings lend support to the androgen theory of ASD, according to which elevated levels of testosterone during fetal development may contribute to the development of ASD. Finally, concerning changes in pigmentation, it is of interest that hypomelanotic diseases usually entail autistic symptoms, as is commonly observed in hypomelanosis of Ito (OMIM#300337; Akefeldt and Gillberg, 1991; von Aster et al., 1997; Gómez-Lado et al., 2004). It has been hypothesized that the comorbidity between hypomelanosis and ASD may result from a deficiency in vitamin D (Eyles, 2010; Bakare et al., 2011). In fact, core symptoms of ASD improve after vitamin D supplementation (Jia et al., 2015). Interestingly, core candidates for the globularization of the AMH skull/brain and the evolution of language-readiness are involved in vitamin D homeostasis and function (see Benítez-Burraco and Boeckx, 2015, for details).

As noted above, regardless of the different selectionist scenarios that may account for the traits commonly found in domesticated mammals, a role for NC hypofunction during embryonic development has been proposed (see Wilkins et al., 2014, for details). No comprehensive view of the role (if any) of the NC in the aetiopathogenesis of ASD has been provided to date. Still, it is important to note that neurocristopathies (that is, conditions resulting from NC defects) commonly involve autistic features. For instance, in CHARGE syndrome (OMIM#214800) autistic traits coexist with developmental abnormalities affecting endocrine, reproductive, urinary and digestive systems, along with skeletal and craniofacial features (Fernell et al., 1999). Given this background, we will now examine whether candidates for ASD are overrepresented among the genes believed to play a central role in NC development and function, with a special emphasis on those that interact with genes important for the globularization of the AMH skull/brain.

## ASD and the genetics of the domestication syndrome

In order to improve our characterization of the domesticated traits in ASD, it is of interest to assess whether candidate genes for this condition (with a particular emphasis on language disabilities) are overrepresented among, or are functionally related to, candidates for domestication. We have relied on an extended list of candidates, which includes the core set of genes proposed by Wilkins et al. (2014), plus a subset of the genes involved in the globularization of the AMH skull/brain and the emergence of language-readiness that are functionally related to them, through direct interaction, and/or that play a role in the development and function of the NC (see Benítez-Burraco et al., in press, for details). Our list also comprises NC-related genes known to play a key role in craniofacial development and/or disorders. As noted above, most of the domesticated traits result from the modification of the cranial region and many of the ASD distinctive features concern the skull, face and brain. Moreover, as reasoned in Boeckx and Benítez-Burraco (2014a,b), we expect that our language-readiness resulted from changes in the development of the skull/brain, but also from the refinement of the externalization devices, specifically, the orofacial region: As also noted above, the impairment of oromotor function has been hypothesized to account for some language deficits in ASD. Table 1 provides a full list and a schematic characterization of these genes.

**Table 1:** **List of putative candidate genes for domestication and ASD**.

**Gene symbol**	**Gene name**	**Domestication^a^**	**Language-readiness^b^**	**NCC^c^**	**Craniofacial^d^**	**Brain rhythmicity^e^**	**ASD**	
							**Candidate^f^**	**Differentially expressed^g^**
***ALX1***	Aristaless-like homeobox protein 1			+	+			
***ALX3***	Aristaless-like homeobox protein 3			+	+			
***ALX4***	Aristaless-like homeobox protein 4			+	+			
***AXIN2***	Axin 2			+	+			+
***BAZ1B***	Bromodomain adjacent to zinc finger domain 1B	**+**		+				
***BMP2***	Bone morphogenetic protein 2	+	+	+	+			
***BMP7***	Bone morphogenetic protein 7	+	+	+				+
***CDC42***	Cell division cycle 42		+	+				+
***CHD7***	Chromodomain helicase DNA binding protein 7	**+**		+			+	+
***CITED2***	Cbp/p300 interacting transactivator with Glu/Asp rich carboxy-terminal domain 2		+	+				+
***CTNNB1***	Catenin Beta 1		+	+			+	+
***DLX1***	Distal-less homeobox 1		+	+				+
***DLX2***	Distal-less homeobox 2	+	+	+				
***DLX5***	Distal-less homeobox 5	+	+	+	+	+	+	
***DLX6***	Distal-less homeobox 6		+	+	+	+	+	
***EDN1***	Endothelin 1			+	+			+
***EDN3***	Endothelin 3	**+**		+				+
***EDNRA***	Endothelin receptor type A			+	+			+
***EDNRB***	Endothelin receptor type B	**+**		+				+
***ERF***	ETS2 repressor factor			+	+			+
***FGF7***	Fibroblast growth factor 7		+	+				
***FGF8***	Fibroblast growth factor 8	**+**	+	+				+
***FGFR1***	Fibroblast growth factor receptor 1	+	+	+	+			
***FGFR2***	Fibroblast growth factor receptor 2			+	+		+	
***FOXD3***	Forkhead box D3	**+**		+			+	
***FOXP2***	Forkhead box P2	+	+					
***FREM1***	FRAS1 related extracellular matrix 1			+	+			
***GDNF***	Glial-derived neurotrophic factor	**+**		+				
***GLI3***	GLI family zinc finger 3	+	+	+	+			
***GRHL3***	Grainyhead like transcription factor 3			+	+			
***GSC***	Goosecoid homeobox			+	+			
***HES1***	Hes family bHLH transcription factor 1	+	+	+				+
***HOXA2***	Homeobox A2			+	+	+		
***HSH2D***	Hematopoietic SH2 domain containing			+	+			
***KIT***	KIT proto-oncogene receptor tyrosine kinase	**+**		+			+	+
***MAGOH***	Mago homolog, exon junction complex core component	**+**		+				+
***MITF***	Microphthalmia-associated transcription factor	**+**		+				+
***MSX1***	Msh homeobox 1			+	+		+	+
***MSX2***	Msh homeobox 2			+	+			+
***NCAM1***	Neural cell adhesion molecule 1	+	+				+	+
***NODAL***	Nodal growth differentiation factor	+	+					+
***NOG***	Noggin			+	+			
***NTN1***	Netrin 1			+	+			+
***PAX3***	Paired box 3	**+**	+	+				
***PAX6***	Paired box 6		+	+		+	+	+
***PAX7***	Paired box 7				+			+
***POLR1A***	Polymerase (RNA) I subunit A				+		+	
***POU3F2***	POU class 3 homeobox 2	+	+				+	+
***PQBP1***	Polyglutamine binding protein 1	+	+					+
***PTCH1***	Patched 1			+	+		+	+
***RET***	Ret proto-oncogene	**+**		+				+
***ROBO1***	Roundabout guidance receptor 1		+	+				+
***ROBO2***	Roundabout guidance receptor 2		+	+			+	
***RUNX2***	Runt related transcription factor 2	+	+	+	+			
***SATB2***	Special AT-rich sequence binding- homeobox 2		+	+				+
***SHH***	Sonic hedgehog	+	+	+	+			
***SIX2***	Sine oculis-related homeobox 2			+	+			
***SLIT1***	Slit guidance ligand 1		+	+				
***SLIT2***	Slit guidance ligand 2		+	+				+
***SOX2***	Sex determining region Y-box 2	**+**	+	+				+
***SOX9***	Sex determining region Y-box 9	**+**	+	+	+		+	+
***SOX10***	Sex determining region Y-box 10	**+**	+	+			+	
***SPECC1L***	Sperm antigen with calponin homology and coiled-coil domains 1-like			+	+			+
***TCF12***	Trascription factor 12			+	+			+
***TCOF1***	Treacle ribosome biogenesis factor 1	**+**		+				
***VCAN***	Versican		+	+				+
***ZIC1***	Zinc finger protein family member 1			+	+			+

a *Core candidates for the “domestication syndrome” according to Wilkins et al. (2014) (bold italicized tags) plus language-readiness genes functionally interacting with them according to Benítez-Burraco et al. (in press) (regular tags).*

b *Genes highlighted as candidates for globularization of the AMH skull/brain and the emergence of language-readiness according to Boeckx and Benítez-Burraco (2014a,b) and Benítez-Burraco and Boeckx (2015).*

c *Involved in neural crest (NC) development and function.*

d *Involved in craniofacial development and/or found mutated in craniofacial syndromes.*

e *Involved in brain oscillation and rhythmicity.*

f *Candidate for ASD as resulting from genomic studies (pathogenic SNPs, association studies, CNVs, functional studies, etc.).*

g *Differentially expressed in postmortem brain tissues of ASD-vs.-control individuals (see text for details).*

When we tried to identify ASD-candidates among this extended list of genes via PubMed (http://www.ncbi.nlm.nih.gov/pubmed), we found out that nearly 25% of them have been suggested to play a role in the aetiopathogenesis of ASD. If we also consider genes that we found differentially expressed in postmortem brain tissues isolated from patients (as discussed in the subsequent section), the percentage rises above 50%. Interestingly, some of these genes are thought to be involved in brain rhythmicity (see Table 1), plausibly contributing to the oscillopathic signature of the ASD brain during language processing.

**Figure 2:**
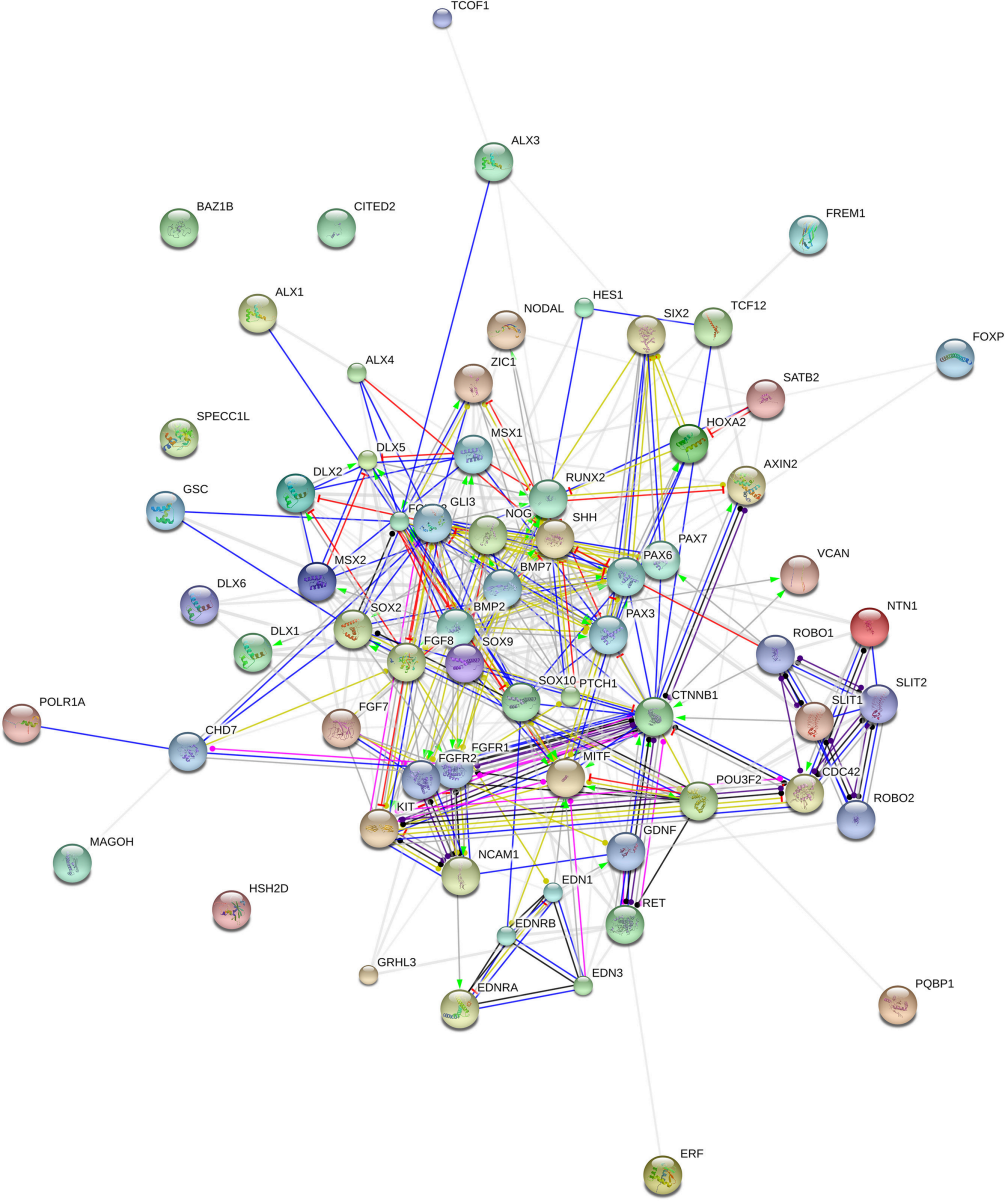
**Functional links among candidates for domestication and ASD**. The chart (drawn using String 10.0 license-free software, http://string-db.org/) shows the network of known and predicted interactions among all genes listed in Table 1. The colored nodes symbolize proteins: small nodes are proteins with unknown 3D structure, while large nodes are those with known 3D structures. The color of the edges represent different kind of known protein-protein associations. Green: activation, red: inhibition, dark blue: binding, light blue: phenotype, dark purple: catalysis, light purple: posttranslational modification, black: reaction, yellow: transcriptional regulation. Edges ending in an arrow symbolize positive effects, edges ending in a bar symbolize negative effects, whereas edges ending in a circle symbolize unspecified effects. Gray edges symbolize predicted links based on literature search ((co-mention in PubMed abstracts). Stronger associations between proteins are represented by thicker lines. The medium confidence value was 0.0400 (a 40% probability that a predicted link exists between two enzymes in the same metabolic map in the KEGG database: http://www.genome.jp/kegg/pathway.html). String 10 predicts associations between proteins that derive from a limited set of databases: genomic context, high-throughput experiments, conserved coexpression, and the knowledge previously gained from text mining (Szklarczyk et al., 2015). This is why the figure does not represent a fully connected graph (evidence for additional links are provided in the main text). Importantly, the diagram only represents the potential connectivity between the involved proteins, which has to be mapped onto particular biochemical networks, signaling pathways, cellular properties, aspects of neuronal function, or cell-types of interest that can be confidently related to aspects of language development and function.

We expect that the genes we highlight here are functionally interconnected and map on to specific pathways, signaling cascades, or aspects of brain development and function, of interest for language processing and the aetiopathology of ASD. *In silico* analyses offer promising insights. Accordingly, String 10 (http://www.string-db.org) predicts quite robust links between most of these genes (Figure 2). Likewise, ontology analyses by Panther (http://www.pantherdb.org) suggest that they might play biological functions important for ASD and be part of signaling pathways known to be impaired in this condition (Table 2).

**Table 2:** **GO classifications of candidates for domestication and ASD**.

**Biological process**	**%^a^**	**Pathway**	
Metabolic process (GO:0008152)	21.60%	Axon guidance mediated by Slit/Robo (P00008)	12.20%
Biological regulation (GO:0065007)	18.80%	TGF-beta signaling pathway (P00052)	10.20%
Developmental process (GO:0032502)	17.00%	Endothelin signaling pathway (P00019)	8.20%
Cellular process (GO:0009987)	13.80%	Gonadotropin releasing hormone receptor pathway (P06664)	8.20%
Multicellular organismal process (GO:0032501)	11.50%	Wnt signaling pathway (P00057)	8.20%
Immune system process (GO:0002376)	4.10%	FGF signaling pathway (P00021)	8.20%
Apoptotic process (GO:0006915)	3.20%	Angiogenesis (P00005)	6.10%
Response to stimulus (GO:0050896)	3.20%	Hedgehog signaling pathway (P00025)	6.10%
Biological adhesion (GO:0022610)	3.20%	Axon guidance mediated by netrin (P00009)	4.10%
Localization (GO:0051179)	1.80%	CCKR signaling map (P06959)	4.10%

a *Numbers refer to percent of gene hit against total of process or pathway hits. Only the top 10 functions, filted after Bonferroni post-hoc correction, have been included.*

### Candidate genes: a functional characterization

Some of Wilkins et al.'s (2014) original candidates for the domestication syndrome are candidates for ASD. *KIT* mutations have been found in patients featuring ASD symptoms (Kilsby et al., 2013). KIT is a tyrosine kinase receptor (Kasamatsu et al., 2008), which acts as a key developmental regulator in the NC-derived processes of hematopoiesis, melanogenesis, and gametogenesis (Rothschild et al., 2003). In rats mutations of *Kit* impair hippocampal synaptic potentiation and spatial learning and memory (Katafuchi et al., 2000). Likewise, whole-genome sequencing analyses have identified deleterious variants of *CHD7* in ASD probands (Jiang et al., 2013). *CHD7* is known to be the main candidate for CHARGE syndrome (Vissers et al., 2004; Lalani et al., 2006), mentioned above. Interestingly, CHARGE syndrome also involves microcephaly, face asymmetry, cleft lip/palate, along with variable degrees of intellectual disability (Pisano et al., 2014; Hale et al., 2016, for review). Changes in the expression pattern of *CDH7* can also result in behavioral anomalies resembling the autistic phenotype. Accordingly, *in utero* exposure to heavy metals in mice increases autism-like behavioral phenotypes in adult animals through inducing the hypomethylation of *Chd7* (Hill et al., 2015). *FOXD3*, encoding a transcription factor, is downregulated by DISC1 (Drerup et al., 2009), a robust candidate for schizophrenia that has been also associated to ASD (Williams et al., 2009; Zheng et al., 2011; Kanduri et al., 2016). *FOXD3* maps within one of the present-day human-specific differentially-methylated genomic regions (DMRs) (Gokhman et al., 2014). Interestingly, loss of *Disc1* results in abnormal NCC migration and differentiation (Drerup et al., 2009). Also, DISC1 downregulates *SOX10*, another NC gene, involved in the maintenance of precursor NCC pools, in the timing of NCC migration onset, and in the induction of their differentiation; it is also implicated in oligodendrocyte differentiation (Hattori et al., 2014). In turn, SOX10 interacts with PAX3, another core candidate proposed by Wilkins et al. (2014), and with POU3F2 (Smit et al., 2000). Sequence and CNVs affecting *POU3F2* have been found in subjects with ASD, and in individuals with different developmental and language delays (Huang et al., 2005; Lin et al., 2011). POU3F2 is a known interactor of *FOXP2*, the renowned “language gene” (Maricic et al., 2013). AMHs bear a derived allele of the binding site which is less efficient in activating transcription than the Neanderthal/Denisovan counterpart (Maricic et al., 2013). Likewise, *POU3F2* has been associated with human accelerated conserved non-coding sequences (haCNSs) (Miller et al., 2014). Also, it interacts with PQBP1, which has been linked to intellectual disability (Wang et al., 2013) and developmental delay and microcephaly (Li et al., 2013). Also *SOX9*, considered a master regulator of craniofacial development and related to several congenital skeletal malformations (Mansour et al., 2002; Gordon et al., 2009; Lee and Saint-Jeannet, 2011), is found among the candidates for ASD.

Accordingly, gene and miRNA expression profiling using cell-line derived total RNA has revealed *SOX9* as one of the genes dysregulated in ASD (Ghahramani Seno et al., 2011). As discussed in detail by Benítez-Burraco et al. (in press) *SOX9* interacts with *BMP2, BMP7, DLX2*, and *HES1*. All of them are core components of the network believed important for globularization and language-readiness (reviewed in Boeckx and Benítez-Burraco, 2014a). In addition, all of them are involved in NCC development and migration, and in the patterning of NC-derived tissues (Mallo, 2001; Gajavelli et al., 2004; Correia et al., 2007; Glejzer et al., 2011; Ishii et al., 2012). *BMP2* is a key osteogenic regulator, which has been associated to craniosynostosis (Justice et al., 2012; Lattanzi et al., 2013). *BMP2, BMP7*, and *DLX2* act upstream *SOX9* (Sperber et al., 2008; Li et al., 2013). In turn, *SOX9* mediates the retinoic acid-induced expression of *HES1*, known also to be involved in language function, craniofacial development, and neuron growth and interconnection (reviewed in Boeckx and Benítez-Burraco, 2014b). Importantly, retinoic acid also regulates the expression of other genes that are relevant for language, like *FOXP2* (Devanna et al., 2014), or for globularization, like *ASCL1* (see Benítez-Burraco and Boeckx, 2015, for details). Retinoic acid has proven to be important for brain plasticity (Luo et al., 2009), and memory and learning processes (Etchamendy et al., 2003; Jiang et al., 2012). Recent whole-exome sequencing analyses have linked retinoic acid regulation pathways to ASD (Moreno-Ramos et al., 2015). In neuronal cells reduced levels of RORA downregulate multiple transcriptional targets that are significantly enriched in biological functions negatively impacted in ASD and which include known ASD-associated genes, like *A2BP1, CYP19A1, ITPR1, NLGN1*, and *NTRK2* (Sarachana and Hu, 2013a). *RORA* itself is downregulated in postmortem prefrontal cortex and cerebellum of subjects with ASD (Nguyen et al., 2010). *RORA* is differentially regulated in them by masculine and feminine hormones: Whereas it is under negative feedback regulation by androgens, it is under positive regulation by estrogens (Sarachana et al., 2011; Sarachana and Hu, 2013b). In certain regions of the brain this sexually dimorphic expression is also found in several of RORA's targets and this correlation is much higher in the cortex of males (Hu et al., 2015). Perhaps not surprisingly, synthetic RORα/γ agonist improve autistic symptoms in animal models of the disease, particularly, repetitive behavior (Wang et al., 2016).

We wish to highlight two other genes thought to be involved in the changes that brought about modern language that are also candidates for ASD and interact with core candidates for the domestication syndrome as posited by Wilkins et al. The first one is *DLX5*, involved in crucial aspects of NC development (McLarren et al., 2003; Ruest et al., 2003), but also of skull and brain development (Kraus and Lufkin, 2006; Wang et al., 2010). Accordingly, it plays a role in thalamic development (Jones and Rubenstein, 2004) and contributes to regulate the migration and differentiation of precursors of GABA-expressing neurons in the forebrain (Cobos et al., 2006). *DLX5* is a candidate for ASD (Nakashima et al., 2010), due to an ultraconserved cis-regulatory element (Poitras et al., 2010), which is bound by GTF2I, encoded by one of the genes commonly deleted in Williams-Beuren syndrome (OMIM#194050) and a candidate for ASD too (Malenfant et al., 2012). Additionally, *DLX5* is regulated by MECP2 (Miyano et al., 2008), encoded by the main candidate for Rett syndrome (OMIM#312750), a condition entailing problems for motor coordination, autistic behavior, and language regression (Uchino et al., 2001; Veenstra-VanderWeele and Cook, 2004). Interestingly, Dlx5/6(±) mice exhibit abnormal pattern of γ rhythms resulting from alterations in GABAergic interneurons, particularly in fast-spiking interneurons (Cho et al., 2015). In addition, DLX5 interacts with key candidates for language evolution, in particular, with *RUNX2* and *FOXP2* (see Boeckx and Benítez-Burraco, 2014a, for details). The second one is *NCAM1*, which is also a target of both RUNX2 (Kuhlwilm et al., 2013) and FOXP2 (Konopka et al., 2009). In mice mutations in the gene affect working/episodic-like memory (Bisaz et al., 2013), whereas overexpression of the Ncam1 extracellular proteolytic cleavage fragment impacts on GABAergic innervation, affecting long- and short-term potentiation in the prefrontal cortex (Brennaman et al., 2011). *NCAM1* encodes a cell adhesion protein involved in axonal and dendritic growth and synaptic plasticity (Rønn et al., 2000; Hansen et al., 2008). It interacts with VCAM1 which is involved in cell adhesion and the control of neurogenesis (Kokovay et al., 2012), and which bears a fixed (D414G) change in AMHs compared to Neanderthals/Denisovans (Pääbo, 2014). *VCAM1* is upregulated by CLOCK, which plays a key role in the modulation of circadian rhythm (Gao et al., 2014). Together with other circadian-relevant genes *CLOCK* seems to be involved in the psychopathology of ASD cases entailing sleep disturbances (Yang et al., 2016). The circadian modulation of synaptic function has been hypothesized to contribute decisively to ASD (Bourgeron, 2007). In turn, *CLOCK* interacts with *RUNX2* and with several other candidates for language-readiness, like *DUSP1*, involved in vocal learning (Doi et al., 2007), and *USF1*, which regulates synaptic plasticity, neuronal survival and differentiation (Tabuchi et al., 2002; Steiger et al., 2004). USF1 binds the promoter of *FMR1* (Kumari and Usdin, 2001), a strong candidate for Fragile-X syndrome (OMIM#300624), which presents with language problems and ASD features (Kaufmann et al., 2004; Smith et al., 2012). The regulatory region of *USF1* shows many fixed or high frequency changes compared to Denisovans (Meyer et al., 2012).

As shown in Table 1, several of the genes important for globularization and language-readiness are involved in NC development and function and some of them are also candidates for ASD. Accordingly, we expect them to contribute to the abnormal domesticated features observed in patients with ASD, and also to their distinctive language profile. Among them we wish mention *CTNNB1, DLX1, DLX6, PAX6*, and *ROBO2*. CTNNB1 is a component of the Wnt/β-catenin signaling pathway, known to be impaired in ASD (Cao et al., 2012; Zhang et al., 2012; Martin et al., 2013). CTNNB1 controls aspects of NC development, from NC induction, lineage decisions, to differentiation (Hari et al., 2012). As noted in Boeckx and Benítez-Burraco (2014b), CTNNB1 is expected to interact with many of the genes highlighted as important for the evolution of language-readiness, specifically with RUNX2 and SLIT2/ROBO1 signals. Regarding DLX1, it is a robust NC marker (Ishii et al., 2012), involved in patterning and morphogenetic processes in NC-derived tissues (Mallo, 2001). It also regulates the development of the skull and the brain (Andrews et al., 2003; Jones and Rubenstein, 2004). In mice *Dlx1* downregulation results in reduced glutamatergic input to the hippocampus (Jones et al., 2011), as well as in changes in interneuron subtypes and migration patterns in the cortex (Ghanem et al., 2008). *DLX1* is found to be downregulated in ASD (Voineagu et al., 2011; McKinsey et al., 2013). ROBO2 is one of the DLX1 interactors. Slit/Robo signaling regulates early NCC migration (Jia et al., 2005) *ROBO2* is also involved in thalamocortical axons (TCA) development, known to be important for the modulation of cognitive functions (López-Bendito et al., 2007; Marcos-Mondéjar et al., 2012). *ROBO2* is a candidate for ASD (Suda et al., 2011), but also for different types of language disorders, like dyslexia (Fisher et al., 2002) and speech-sound disorder and reading (Stein et al., 2004). It has been related as well to expressive vocabulary growth in the normal population (St Pourcain et al., 2014). Finally, *PAX6* controls the migration of NCCs from the anterior midbrain (Matsuo et al., 1993). *PAX6* is involved as well in the development of the brain (Valverde et al., 2000; Tyas et al., 2003; Caballero et al., 2014). Mutations on *PAX6* have been reported in some forms of ASD (Maekawa et al., 2009), although they also impact in working memory (Bamiou et al., 2007). Alterations of *PAX6* expression in the brain of people with ASD may account for the observed imbalance in excitatory/inhibitory neuronal activity (Kim et al., 2014). And like many of the genes reviewed above, *PAX6* is functionally related to both *FOXP2* and *RUNX2*, and it also targets *POU3F2* (see Benítez-Burraco and Boeckx, 2015, for details).

Most of the NCC-genes mentioned here are known to play a key role in the development and patterning of the craniofacial complex, and to be associated to congenital craniofacial defects (Table 1) (see Twigg and Wilkie, 2015 for review). Many of these genes are known candidates for ASD, including *DLX5* and *DLX6* (reviewed above), *FGFR2, MSX1, POLR1A*, and *PTCH1*. Both *DLX5* and *DLX6* are indeed required for NC-derived facial morphogenesis (Gitton et al., 2011) FGFRs are among the main craniosynostosis-associated genes. In particular, gain-of-function mutations in *FGFR2* are typically associated to Apert (OMIM#101200) and Crouzon (OMIM#123500) syndromes, while both *FGFR1* and *FGFR2* are found mutated in Pfeiffer syndrome (OMIM#101600) (Lattanzi et al., 2012). All these syndromic craniosynostoses occasionally present with variable degree of ASD-like mental retardation (Morey-Canellas et al., 2003). *MSX1* encodes a transcriptional repressor involved in craniofacial development and shaping (particularly in odontogenesis) (Alappat et al., 2003; Lattanzi, forthcoming). It is expressed in the NC (Khadka et al., 2006), where it acts as a master regulator of gene expression (Attanasio et al., 2013). Although it has not been associated to ASD, *MSX1* is a direct downstream target of DLX5 during early inner ear formation (Sajan et al., 2011). The gene is also a critical intrinsic dopaminergic neuron determinant (Andersson et al., 2006) and is found mutated in some patients with Wolf-Hirschhorn syndrome (OMIM#194190), a clinical condition entailing profound mental retardation and craniofacial dysmorphism (Campbell et al., 1989). *POLR1A*, found mutated in acrofacial dysostosis (Cincinnati type, OMIM#616462) involving microcephaly, plays a role in the regulation of NC-derived skeletal precursor cells (Weaver et al., 2015). In some ASD subjects CNVs result in fusion transcripts involving *POLR1A*, although no fusion transcripts have been detected to date (Holt et al., 2012).

Finally, it is worth mentioning that genes encoding primary cilium signaling molecules, such as *SHH, GLI3*, and *PTCH1*, are all primarily involved in congenital malformations affecting the midline craniofacial compartment (Brugmann et al., 2010; Rice et al., 2010). Specifically, *PTCH1* is required in the NC-dependent orofacial development and gives rise to orofacial clefting, when mutated (Metzis et al., 2013). Heterozygous mutations of either *SHH* or *PTCH1* are typically found in holoprosencephaly (OMIM#610828, and #236100), a genetically heterogeneous, highly prevalent congenital forebrain anomaly in humans, associated with mental retardation and craniofacial malformations (Ming et al., 2002; Mercier et al., 2011). In addition, a 22-bp deletion in this gene has been found in a girl with ASD and Gorlin syndrome, a complex condition involving macrocrania and hypertelorism (Delbroek et al., 2011).

### Candidate genes: expression profiles in the ASD brain

**Figure 3:**
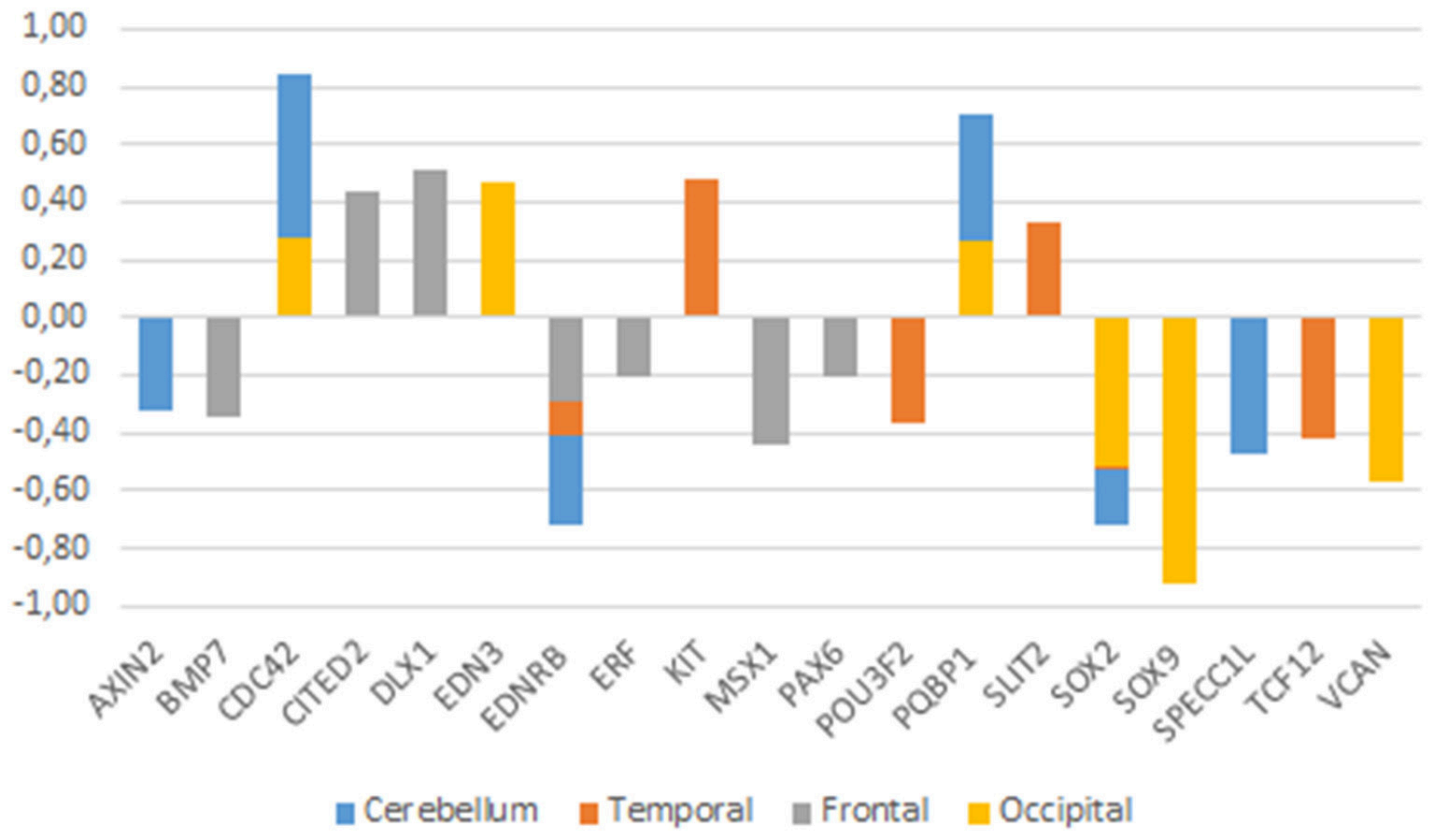
**Expression profiles of candidate genes in the ASD brain**. Data were gathered from the following microarray expression datasets available on the Gene Expression Omnibus database (GEO datasets, http://www.ncbi.nlm.nih.gov/gds): GSE28521 (Voineagu et al., 2011) for the temporal and frontal cortices, GSE38322 (Ginsberg et al., 2012) for the cerebellum and the occipital cortex. Data are shown as log transformation of fold changes (logFC) between patients and corresponding controls. Only genes showing statistically significant (*p* < 0.05) differential expression were considered. Additional details may be found in the Supplemental information file.

If our hypothesis is on the right track, we expect that the genes we highlight here are dysregulated in the brain of people with ASD, particularly in areas important for language processing. Accordingly, we surveyed the Gene Expression Omnibus (GEO) repository (https://www.ncbi.nlm.nih.gov/gds) searching for their expression profiles in the cerebellum and the temporal cortex (but also in the frontal and occipital cortices) in patients with ASD. This should help identify new candidates for ASD in the context of domestication and language-readiness (Table 1). Overall, we could find significant expression values for some of our candidates and learnt that they are up- or downregulated in the brain of autists (Figure 3).

Among the genes that are significantly downregulated in the cerebellum we found *AXIN2, EDNRB, SOX2, SPECC1L, TCF12*, and *VCAN*, whereas *CDC42*, and *PQBP1* are found upregulated in this region (Figure 3). Although none of them has been associated to ASD, they stroke us as promising candidates for the atypical presentation of the domestication syndrome in ASD. *AXIN2* is expressed in the cranial NC and is needed for NC-derived frontal bone osteogenesis (Yu et al., 2005; Li et al., 2015a). This gene is also expressed as a specific marker for suture stem cells (Maruyama et al., 2016), but also acts as a negative regulator of canonical Wnt pathway, contributing to the stability of CTNNB1 (Li et al., 2015a). Speech alterations are also observed in people with *AXIN2* mutations causing non-syndromic oligodontia (Liu et al., 2015). *EDNRB* encodes a receptor for endothelins, known to be potent vasoactive peptides. Mutations in this gene are associated to increased susceptibility to Hirschsprung disease (OMIM#600155), a neurocristopathy characterized by congenital absence of intrinsic ganglion cells in the enteric nervous plexa (Amiel et al., 2008). Waardenburg syndrome (OMIM#277580), a genetically heterogeneous condition which may involve developmental delay subsidiary to sensorineural hearing loss, has also been associated with mutations in *EDNRB* (Read and Newton, 1997). *SOX2*, one of core candidates for domestication (Wilkins et al., 2014), encodes an interactors of the GLI factors as part of the SHH-GLI signaling pathway involved in NCC fate (Oosterveen et al., 2012, 2013; Peterson et al., 2012), but also in the globularization of the AMH skull/brain (see Boeckx et al., submitted for details). SOX2 interacts as well with the BMP signaling (Li et al., 2015b). Interestingly, SOX2 regulates *PQBP1*, highlighted above as one of POU3F2 interactors. *SPECC1L* is found mutated in Opitz G/BBB syndrome (OMIM# #145410) and in facial clefting (Kruszka et al., 2015). This gene functions in NC development (Wilson et al., 2016) and is specifically involved in facial morphogenesis (Saadi et al., 2011). *TCF12* is highly expressed in embryonic precursors of skull/brain structures, including NC-derived head mesenchyme (Uittenbogaard and Chiaramello, 2002). *TCF12* directly interacts with *TWIST1*, mutated in Saethre-Chotzen syndrome (OMIM#601622), which features complex craniosynostosys with variable degrees of intellectual disability, including ASD traits (Maliepaard et al., 2014). Indeed, loss-of-function mutations of *TCF12* have been identified in patients with coronal synostosis, which sometimes involves intellectual disability (Sharma et al., 2013; di Rocco et al., 2014; Paumard-Hernández et al., 2015; Piard et al., 2015). *VCAN* encodes versican-1, a protein that guides migratory NCCs (Dutt et al., 2006) and which shows a fixed N3042D change in AMHs (Pääbo, 2014). Finally, *CDC42* controls NC stem cell proliferation (Fuchs et al., 2009). Inactivation of Cdc42 in NCCs causes craniofacial and cardiovascular morphogenesis defects (Liu et al., 2013). As discussed in detail in Boeckx and Benítez-Burraco (2014b) *CDC42* is an important gene regarding language evolution, because of its functional connections with core candidates for globularization and the externalization of language (including *FOXP2, RUNX2, SLIT2*, and *ROBO1*), with genes related to language disorders (like *CMIP*), and with genes known to have changed after our split from Neanderthals and Denisovans (like *ITGB4, ARHGAP32, ANAPC10*, and *CDC42EP4*).

With regards to the temporal cortex, we found that *EDNRB, POU3F2, SOX2, VCAN*, and *TCF12* are downregulated in subjects with ASD, whereas *KIT, PQBP1*, and *SLIT2* are upregulated in them (Figure 3). As noted above, *POU3F2, SOX2, KIT* are known candidates for ASD. We have already reviewed all these genes. Concerning the frontal cortex we found that *BMP7, EDNRB, PAX6, ERF*, and *MSX1* are significantly downregulated, whereas *CITED2* and *DLX1* are significantly upregulated (Figure 3). As noted in Table 1, *DLX1, PAX6*, and *MSX1* have been previously associated to ASD. Most of these genes have been already reviewed. *BMP7* is a NCC gene involved in regulation of osteogenesis (Cheng et al., 2003; Anderson et al., 2006) and in skull and brain development (Yuge et al., 2011; Segklia et al., 2012). *BMP7* is also closely related to some of the core candidates for globularization and language- readiness, like *BMP2, DLX1, DLX2*, and *RUNX2*. BMP7 is predicted (according to String 10) to interact with SOX2 via NOG, involved in dopamine neuron production and an inhibitor of BMP signaling (Chiba et al., 2008). Developmental delay and learning disabilities are commonly observed in people with mutations in *BMP7* (Wyatt et al., 2010). *ERF* encodes a member of the ETS family of transcription factors, expressed in migratory cells, including NCCs (Paratore et al., 2002). *ERF* haploinsufficiency gives rise to either coronal or multisuture synostosis, midface hypoplasia, often associated with behavioral and learning difficulties (Twigg et al., 2013). Concerning *CITED2*, this is a functional partner of both FOXP2 and RUNX2 (Luo et al., 2005; Vernes et al., 2011; Nelson et al., 2013), two important genes for the emergence of modern language. *CITED2* is also involved in craniofacial development (Bhattacherjee et al., 2009) and in the establishment of left-right axis through interactions with the BMP signaling and Nodal (Preis et al., 2006; Lopes Floro et al., 2011). Interestingly, 99% of AMHs bear a highly disruptive intergenic change near *CITED2* compared to Altai Neanderthals and Denisovans (Prüfer et al., 2014).

Finally, regarding the occipital cortex, we found that *EDN3, CDC42*, and *PQBP1* are significantly upregulated in ASD, whereas *SOX2, SOX9*, and *VCAN* are downregulated. We have already considered all these genes, with the exception of *EDN3*. This gene encodes an endothelium-derived vasoactive peptide which binds the product of *EDNRB*, playing a key role in the development of neural crest-derived cell lineages, such as melanocytes and enteric neurons. Although *EDN3* is a candidate for Waardenburg syndrome and Hirschsprung disease, the gene has been found significantly dysregulated in children with ASD (Glatt et al., 2011).

As shown in Figure 3, genes that exhibit significant changes in their expression levels in the ASD brain are consistently down- or upregulated across all the regions under analysis. Considering their functions and the phenotypes resulting from their mutation, the most promising of these genes are *CDC42* and *PQBP1* (which are upregulated) and *EDNRB* and *SOX2* (which are found downregulated). As noted above, to date none of them have been associated to ASD, but they emerge as reasonably involved in the pathogenesis of this condition.

**Figure 4:**
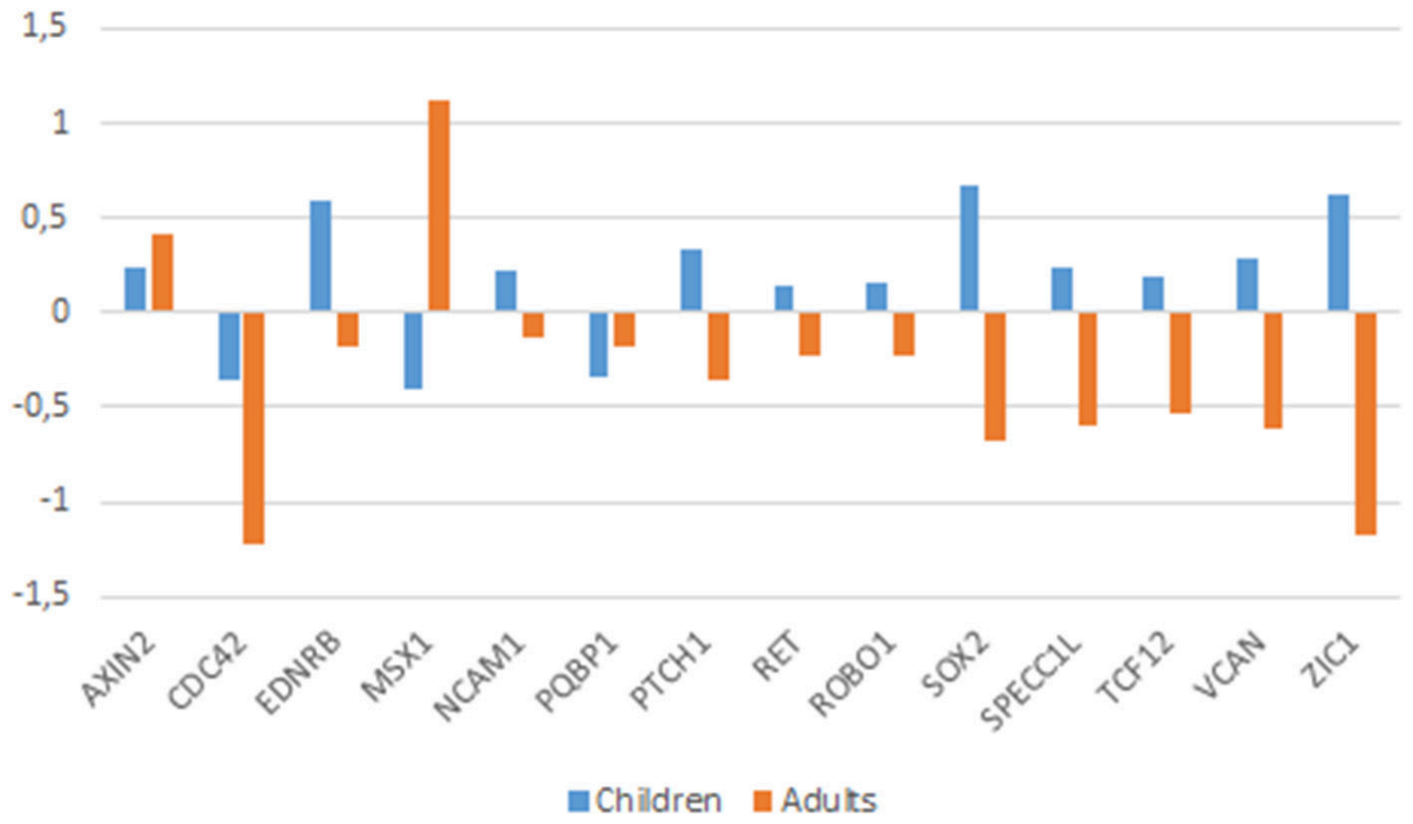
**Expression profiles of candidates genes in the cerebellum of children and adults with ASD**. Expression data were obtained from the microarray expression dataset GSE38322 (Ginsberg et al., 2012) available on the Gene Expression Omnibus database (GEO datasets, http://www.ncbi.nlm.nih.gov/gds). Data are shown as log transformation of fold changes (logFC) between patients and corresponding controls. Only genes showing statistically significant (*p* < 0.05) differential expression were considered. Additional details may be found in the Supplemental information file.

Noteworthy age-related differences in the intrinsic functional connectivity of the brain are observed in ASD: adult patients show reduced connectivity while children tend to exhibit an increased connectivity (Uddin et al., 2013). Therefore, we have further analyzed ASD brain expression data in an age-matched fashion. Due to the available sample characteristics, only expression data obtained from the cerebellum could be analyzed (see Supplemental file for further details). The reduction of the age-related bias in the patients-vs.-controls comparison, enabled finding a higher number of statistically significant dysregulated genes in the ASD brain. Accordingly, we found that in the cerebellum of ASD children (below 11 years old), *CDC42, MSX1, MSX2, NODAL, PQBP1*, and *SLIT2* were downregulated, whereas, *AXIN2, CHD7, CITED2, EDNRB, FGF8, NCAM1, PAX7, PTCH1, RET, ROBO1, SOX2, SPECC1L, TCF12, VCAN*, and *ZIC1* were upregulated. In turn, in the cerebellum of adult patients (aged 22–60 years) we found that *CDC42, CTNNB1, DLX1, EDNRA, EDNRB, HES1, KIT, MAGOH, MITF, NCAM1, NTN1, POU3F2, PQBP1, PTCH1, RET, ROBO1, SATB2, SOX2, SPECC1L, TCF12, VCAN*, and *ZIC1* are downregulated, whereas only *AXIN2, CTNNB1, DLX1, EDN1*, and *MSX1* are upregulated. Overall, we concluded that nearly one third of the candidates for domestication are dysregulated in the cerebellum of people with ASD, and that more than a half are specifically dysregulated in the cerebellum of either children and/or adults with this condition. Genes that are dysregulated in both children and adults with ASD can be regarded as significant contributors to the atypical presentation of the domestication syndrome in this condition. This list encompasses 14 genes: *AXIN2, CDC42, EDNRB, MSX1, NCAM1, PQBP1, PTCH1, RET, ROBO1, SOX2, SPECC1L, TCF12, VCAN*, and *ZIC1*. Interestingly, they exhibit opposite expression profiles in children and adults with ASD (Figure 4). Most of these genes have been already discussed here. In addition, *RET*, encoding a cadherin that plays a crucial role in NC development, is a candidate for Hirschsprung disease (OMIM# 142623; Edery et al., 1994) and is found to be differentially expressed after RUNX2 transfection in neuroblastoma cells (Kuhlwilm et al., 2013). *RET* is downstream *ASCL1* (another candidate for Hirschsprung disease) in noradrenergic brain stem neurons important for respiratory rhythm modulation (Dauger et al., 2001). Likewise, *ZIC1* is needed for NC development (along with *PAX3*) and plays a key role in craniofacial development (Milet et al., 2013; Plouhinec et al., 2014). Mutations in *ZIC1* result in severe coronal synostosis associated with learning difficulties (Twigg et al., 2015).

In the last section of the paper we attempt refining our characterization of ASD as an atypically-domesticated phenotype. In doing so, we will focus on brain function, with a special emphasis on brain oscillations. Accordingly, we will compare the oscillopathic profile of people with ASD during language processing with the oscillatory signature of the TD population and that of non-domesticated primates. Additionally, we will examine the expression profile in the primate brain of the candidate genes we highlight here.

## ASD and wild primates: from brain oscillation to gene expression patterns

### ASD and primate oscillomes

As we have discussed in detail in Benítez-Burraco and Murphy (2016) language impairment in ASD (as ASD itself) can be satisfactorily characterized as an oscillopathic condition. With cognitive disorders exhibiting disorder-specific abnormal oscillatory profiles, it is also noteworthy that species-specific oscillatory patterns seemingly emerge as slight variations within the network constellation that constitutes a universal brain syntax (Buzsáki and Watson, 2012; Buzsáki et al., 2013).

The differences in brain capacity between domesticated and non-domesticated animals (Wilkins et al., 2014) would be predicted to give rise to a corresponding alteration in oscillatory properties (i.e., features of neural oscillations which form part of an individual's “oscillome,” as it is termed in Murphy and Benítez-Burraco, in press; Murphy, 2016a,b). Although we feel that current knowledge is too scarce to permit any reasonable linking hypotheses between the primate and ASD oscillomes, we would like to briefly sketch out a possible route to increasing our understanding of the neural signature of domestication (and failed domestication itineraries).

**Figure 5:**
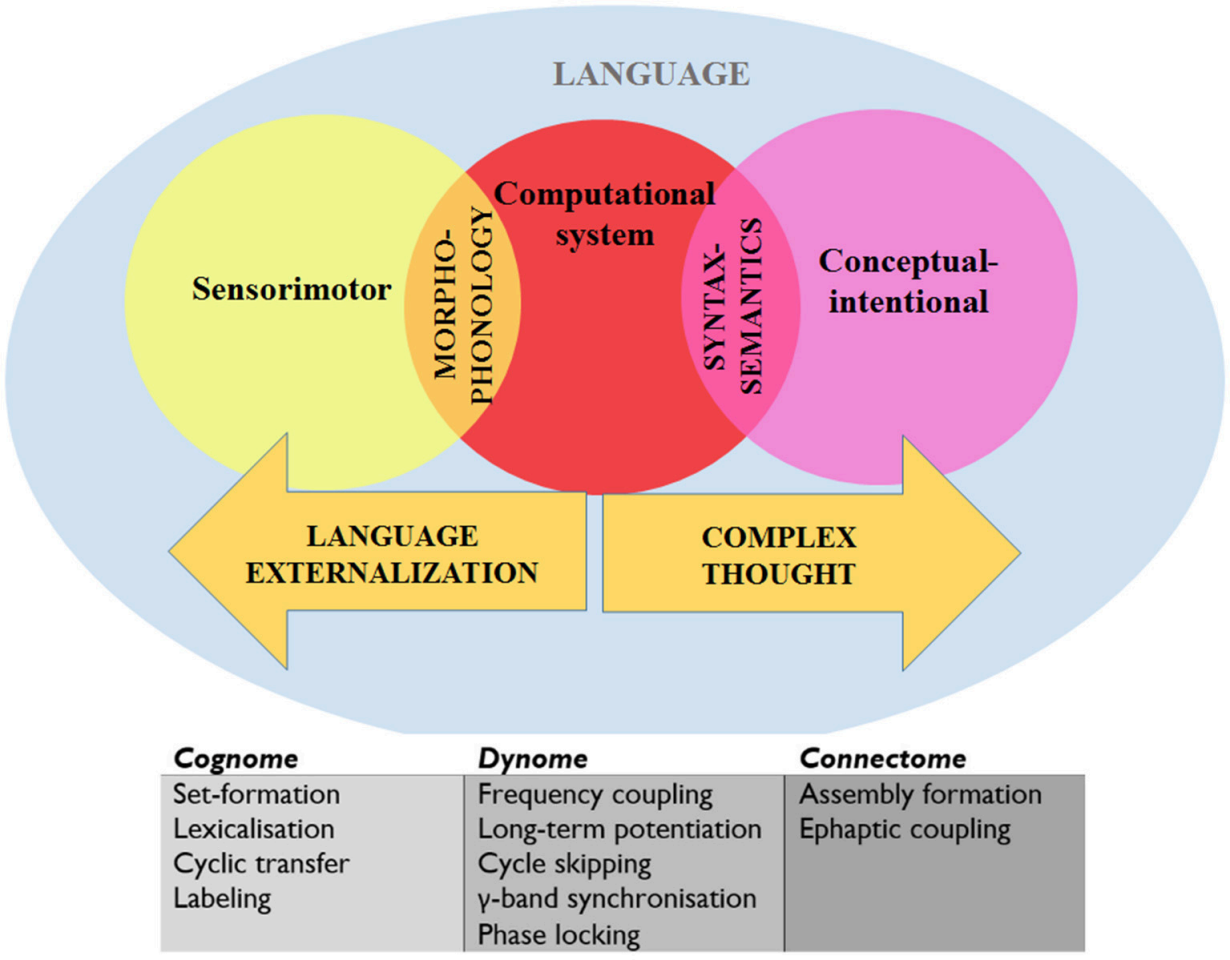
**A schematic view of language representing the systems and interfaces of interest and the levels of analysis**. “Cognome” refers to the operations available to the human nervous system (Poeppel, 2012) and “dynome” refers to brain dynamics (Kopell et al., 2014; Murphy and Benítez-Burraco, in press). See text for details.

Call vocalizations have been found not to be impaired when the homolog of Broca's region in the monkey brain is lesioned, which suggests that other area (like the limbic system and brainstem) are involved (Sage et al., 2006). However, macaques appear to share similar call comprehension substrates with human language comprehension in the left posterior temporal gyrus (Heffner and Heffner, 1986). It would be of interest, for instance, to compare the rhythmic properties of this region of the TD brain with those of the primate brain to see if any particular activity (e.g., coupling and synchronization) is marked in humans. This would also yield insights into how the primate call comprehension system “interfaces” with other cognitive systems (given the appropriate experimental environment), and would also permit the exploration of similar interface properties of human language comprehension, which requires the transfer of information to two interfaces (Figure 5).

Discounting work on evoked potentials (which is itself fairly modest), there are currently only a handful of empirical studies of the monkey oscillome. Brincat and Miller (2015), for instance, discovered functional differences and frequency-specific interactions between the Rhesus hippocampus (HPC) and prefrontal cortex (PFC) during object pair association learning. θ synchrony was found to be greater after errors and decreased after learning; correct associations increased β-α synchrony, which was also greater in the HPC-PFC direction. Esghaei et al. (2015) also suggested that the macaque visual cortex employs phase-amplitude coupling to regulate inter-neuronal correlations, and so the potential for generic oscillomic processes to yield insights into cognitive (dys)function seems apparent. During the internally monitored continuation phase of a synchronization-continuation task, β also appears to increase in Rhesus monkeys (Bartolo and Merchant, 2015), suggesting that—as in humans (Murphy, 2015a)—β is responsible for maintaining the existing cognitive set in memory. β is also involved in “the attentive state and external cues as opposed to detailed muscle activities” in Japanese monkeys (*Macaca fuscata*) (Watanabe et al., 2015). Finally, pulvinar γ is involved in feedforward processing for snake images, and also in cortico-pulvinar-cortical integration for face images (Le et al., 2016), while Ramirez-Villegas et al.'s (2015) study of the macaque hippocampal CA3-CA1 network pointed to the role of γ in memory reactivation, transfer and consolidation.

Currently, there are no studies comparing the oscillome of domesticated and non-domesticated primates, but our prediction would be that non-domesticated primates display a degree of oscillomic difference with domesticated primates comparable to the difference between TD individuals and people with ASD. Table 3 summarizes existing knowledge of the ASD and primate oscillome during a range of cognitive tasks, and it is hard to find any correlations or connections between the two. However, we feel that comparatively exploring these oscillomes will permit a greater understanding of the atavistic neural oscillations of the non-domesticated human and primate brains. Future research should also seek to compare the oscillomes of domesticated and non-domesticated primates in an effort to investigate neural signatures of domestication.

**Table 3:** **Summary of the patterns of rhythmicity observed in wild primates and the observed oscillomic differences in ASD compared to TD subjects**.

**Frequency band**	**Oscillomic monkey profile**	**Oscillopathic profile of autism spectrum disorder**
Delta (~0.5–4 Hz)	Decreased phase-amplitude coupling with γ yields increased visual attention, suggesting that cross-frequency coupling suppression modulates attention.	Increased in eyes-closed resting state exam; predicted to be disrupted in processing phrases involving raising and passives.
Theta (~4–10 Hz)	Decreased phase-amplitude coupling with γ yields increased visual attention; greater HPC-PFC synchrony after object pair association errors.	Reduced cross-frequency coupling with γ; does not synergistically engage with γ during speech; predicted to be disrupted in certain memory retrieval processes.
Alpha (~8–12 Hz)	Increased synchrony with β during correct object pair associations.	Reduced cross-cortically; reduced resting-state α-γ phase amplitude coupling; increased in resting state; predicted to be disrupted during certain lexicalizations.
Beta (~10–30 Hz)	Increased synchrony with α during object pair associations; increases during continuation phase of a synchronization-continuation task.	Reduced in picture-naming tasks; predicted to be disrupted in the maintenance of syntactic objects in raising, passives and *wh*-questions.
Gamma (~30–100 Hz)	Involved in processing snake and face images increases during action sequence updating and memory consolidation, reactivation, and transfer.	Over-connectivity gives rise to increased γ; reduced in rSTG and lIFG during picture naming; predicted to be disrupted quite generally in linguistic cognition.

### Candidate genes: expression profiles in the primate brain

**Figure 6:**
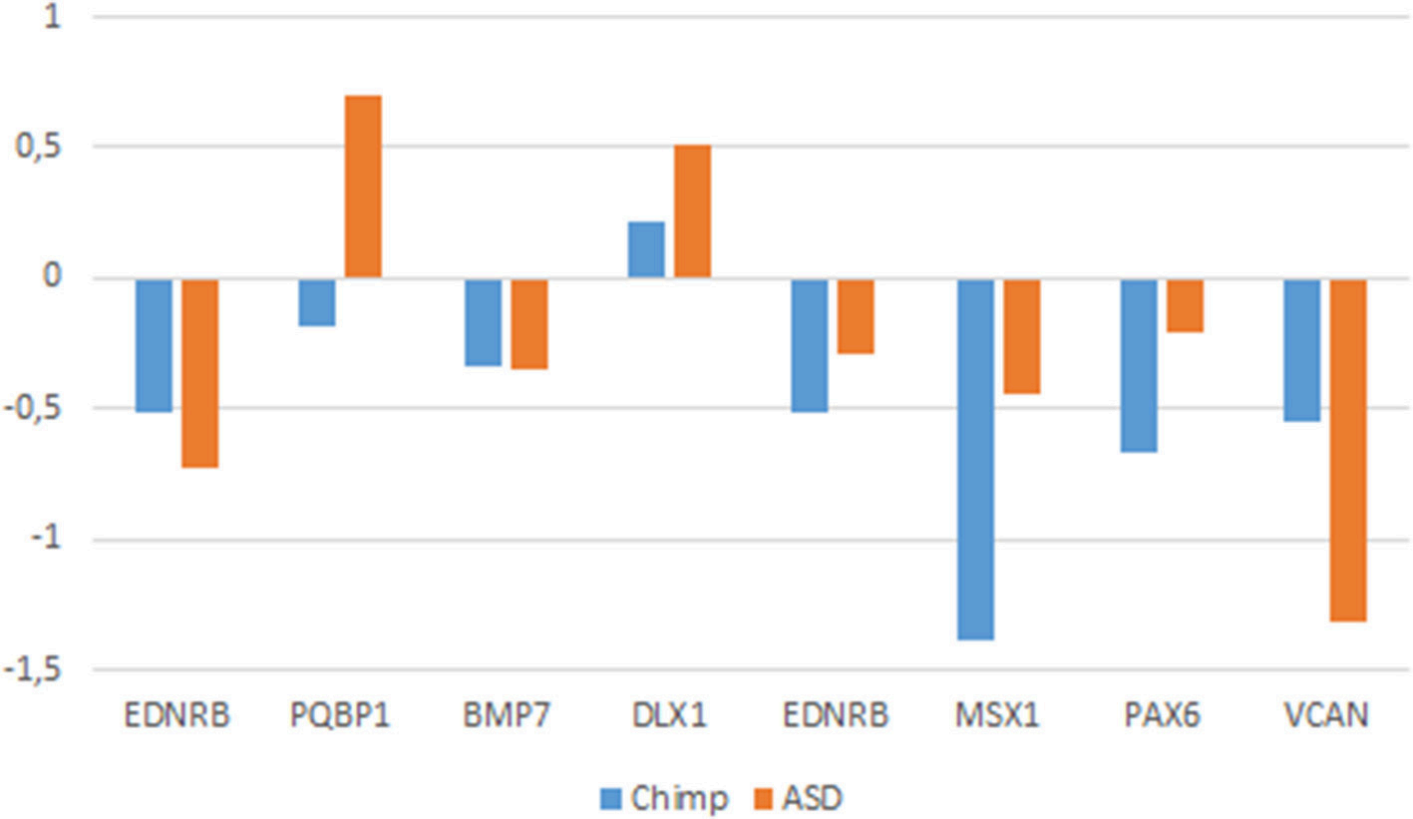
**Comparative expression profiles in chimpanzees and subjects with ASD of candidate genes**. Data were obtained from microarray expression datasets available on the Gene Expression Omnibus database (GEO datasets, http://www.ncbi.nlm.nih.gov/gds): GSE28521 (Voineagu et al., 2011) for the temporal and frontal cortices, and GSE38322 (Ginsberg et al., 2012) for the cerebellum of subjects with ASD; GSE22569 (Somel et al., 2011; Liu et al., 2012) for the cerebellum, GSE18142 (Konopka et al., 2009) for the frontal cortex, and GSE7540 (Cáceres et al., 2003) for the temporal cortex of chimps. Data are shown as log transformation of fold changes (logFC) between patients and corresponding controls. Only genes showing statistically significant (*p* < 0.05) differential expression were considered. Additional details may be found in the Supplemental information file. Note that the plot is intended to display the overall trend of gene expression, given that the relative expression values (i.e., logFC) were obtained from comparative analyses performed on different datasets (based on different designs, samples, and batches).

If our hypothesis turns to be on the right track, we further expect that the genes that we have found dysregulated in the brain of people with ASD show similar expression profiles in conditions where normal socialization failed to occur. Because feral children are scarce and not easily available we examined the expression profiles of these genes in wild primates (chimps). In particular, we selected available gene expression profiles obtained from chimp brain areas that are known to be involved in language processing in humans (the cerebellum, the temporal cortex, and the frontal cortex), as we did for people with ASD. We learnt that most of the genes that we had previously found differentially expressed in the ASD brain data exhibit the same expression pattern in the chimp brain, including *EDNRB* (in the cerebellum), *BMP7, DLX1, EDNRB, MSX1*, and *PAX6* (in the frontal cortex), and *VCAN* (in the temporal cortex) (Figure 6).

## Conclusions

Socialization is a crucial step needed for the achievement of many cognitive abilities that are a signature of the human condition. Language is one of the most prominent of such abilities. Several high prevalent pathological conditions impact on human-specific cognitive abilities, including schizophrenia and ASD. In ASD, social abilities are seriously compromised, but other core cognitive skills, including language, also exhibit differences with the non-affected population. ASD is a multifactorial condition, with wide clinical and genetic heterogeneity. It is still not clear how ASD features emerge from genomic and/or environmental cues during development. In this paper we have focused on language deficits in ASD, although we expect that the lessons we draw here contribute to shedding light on the whole profile of this cognitive condition. In doing so we have adopted and evolutionary perspective, because of the robust link that exists between (abnormal) development and evolution. As we have shown, domesticated traits are absent or are attenuated in people with ASD, and genes that we believe important for the (self)domestication of our species and the evolution of our distinctive cognitive abilities (including language) show abnormal expression patterns in the brains of people with autism. What is more: abnormalities can be traced to the time window when crucial brain rewiring occurs during language acquisition and when changes in the normal configuration of the brain occurs in children with ASD. Additionally, some features of the ASD phenotype can be found (or expected to be found) in wild primates. On the whole, we think that our approach can help illuminate the etiology of ASD primarily because provides robust links between the genome and the environment, and between development and evolution, in line with the current evo-devo approaches to cognitive diseases (see Benítez-Burraco, 2016a, for review). In this sense, the putative involvement of the neural crest in the aetiopathogenesis of ASD emerges as a promising avenue for future research. At the same time, we expect that our approach with help illuminate the evolutionary history of our language-readiness: Our results support the view that language evolution benefitted from a favorable social context that may have resulted from our (self)-domestication.

## Author contributions

AB contributed to all Sections and drafted the manuscript, WL contributed to Sections “Domestic Traits in the ASD Phenotype” and “ASD and the Genetics of the Domestication Syndrome” and revised the manuscript, EM contributed to Sections “Introduction” and “ASD and Wild Primates: from Brain Oscillation to Gene Expression Patterns” and revised the manuscript.

### Conflict of interest statement

The authors declare that the research was conducted in the absence of any commercial or financial relationships that could be construed as a potential conflict of interest.

## References

[B1] Akefeldt A. Gillberg C. (1991). Hypomelanosis of Ito in three cases with autism and autistic-like conditions. Dev. Med. Child Neurol. 33, 737–743. 10.1111/j.1469-8749.1991.tb14953.x 1717328

[B2] Alappat S. Zhang Z. Y. Chen Y. P. (2003). Msx homeobox gene family and craniofacial development. Cell Res. 13, 429–442. 10.1038/sj.cr.7290185 14728799

[B3] Aldridge K. George I. D. Cole K. K. Austin J. R. Takahashi T. N. Duan Y.. (2011). Facial phenotypes in subgroups of prepubertal boys with autism spectrum disorders are correlated with clinical phenotypes. Mol. Autism. 2:15. 10.1186/2040-2392-2-15 21999758 PMC3212884

[B4] Amiel J. Sproat-Emison E. Garcia-Barceo M. Lantieri F. Burzynski G. Borrego S.. (2008). Hirschsprung disease: associated syndromes and genetics: a review. J. Med. Genet. 45, 1–14. 10.1136/jmg.2007.053959 17965226

[B5] Anderson R. M. Stottmann R. W. Choi M. Klingensmith J. (2006). Endogenous bone morphogenetic protein antagonists regulate mammalian neural crest generation and survival. Dev. Dyn. 235, 2507–2520. 10.1002/dvdy.20891 16894609 PMC6626635

[B6] Andersson E. Tryggvason U. Deng Q. Friling S. Alekseenko Z. Robert B.. (2006). Identification of intrinsic determinants of midbrain dopamine neurons. Cell 124, 393–405. 10.1016/j.cell.2005.10.037 16439212

[B7] Andrews G. L. Yun K. Rubenstein J. L. Mastick G. S. (2003). Dlx transcription factors regulate differentiation of dopaminergic neurons of the ventral thalamus. Mol. Cell Neurosci. 23, 107–120. 10.1016/S1044-7431(03)00016-2 12799141

[B8] Attanasio C. Nord A. S. Zhu Y. Blow M. J. Li Z. Liberton D. K.. (2013). Fine tuning of craniofacial morphology by distant-acting enhancers. Science 342:1241006. 10.1126/science.1241006 24159046 PMC3991470

[B9] Bailey A. Phillips W. Rutter M. (1996). Autism: towards an integration of clinical, genetic, neuropsychological, and neurobiological perspectives. J. Child Psychol. Psychiatry 37, 89–126. 10.1111/j.1469-7610.1996.tb01381.x 8655659

[B10] Bakare M. O. Munir K. M. Kinney D. K. (2011). Association of hypomelanotic skin disorders with autism: links to possible etiologic role of vitamin-D levels in autism? Hypothesis 9:e2. 10.5779/hypothesis.v9i1.200 21949515 PMC3177756

[B11] Bamiou D. E. Campbell N. G. Musiek F. E. Taylor R. Chong W. K. Moore A.. (2007). Auditory and verbal working memory deficits in a child with congenital aniridia due to a PAX6 mutation. Int. J. Audiol. 46, 196–202. 10.1080/14992020601175952 17454233

[B12] Bartolo R. Merchant H. (2015). β oscillations are linked to the initiation of sensory-cued movement sequences and the internal guidance of regular tapping in the monkey. J. Neurosci. 35, 4635–4640. 10.1523/JNEUROSCI.4570-14.2015 25788680 PMC6605135

[B13] Belmonte M. K. Saxena-Chandhok T. Cherian R. Muneer R. George L. Karanth P. (2013). Oral motor deficits in speech-impaired children with autism. Front. Integr. Neurosci. 7:47. 10.3389/fnint.2013.00047 23847480 PMC3696837

[B14] Benayed R. Gharani N. Rossman I. Mancuso V. Lazar G. Kamdar S.. (2005). Support for the homeobox transcription factor gene ENGRAILED 2 as an autism spectrum disorder susceptibility locus. Am. J. Hum. Genet. 77, 851–868. 10.1086/497705 16252243 PMC1271392

[B15] Benítez-Burraco A. (2016a). A biolinguistic approach to language disorders: towards a paradigm shift in clinical linguistics, in Advances in Biolinguistics: The Human Language Faculty and Its Biological Basis, eds Boeckx C. A. Fujita K. (London: Routledge), 256–272.

[B16] Benítez-Burraco A. (2016b). A biolinguistic approach to sign languages, in The Oxford Handbook of Deaf Studies: Language and Language Development, eds Marschark M. Spencer P. E. (Oxford: Oxford University Press), 247–263.

[B17] Benítez-Burraco A. Boeckx C. (2015). Possible functional links among brain- and skull-related genes selected in modern humans. Front. Psychol. 6:794. 10.3389/fpsyg.2015.00794 26136701 PMC4468360

[B18] Benítez-Burraco A. Murphy E. (2016). The oscillopathic nature of language deficits in autism: from genes to language evolution. Front. Hum. Neurosci. 10:120. 10.3389/fnhum.2016.00120 27047363 PMC4796018

[B19] Benítez-Burraco A. Theofanopoulou C. Boeckx C. (in press). Globularization domestication. Topoi.. 10.1007/s11245-016-9399-7 Available online at: http://link.springer.com/article/10.1007/s11245-016-9399-7

[B20] Bhattacherjee V. Horn K. H. Singh S. Webb C. L. Pisano M. M. Greene R. M. (2009). CBP/p300 and associated transcriptional co-activators exhibit distinct expression patterns during murine craniofacial and neural tube development. Int. J. Dev. Biol. 53, 1097–1104. 10.1387/ijdb.072489vb 19598128 PMC2746635

[B21] Bisaz R. Boadas-Vaello P. Genoux D. Sandi C. (2013). Age-related cognitive impairments in mice with a conditional ablation of the neural cell adhesion molecule. Learn. Mem. 20, 183–193. 10.1101/lm.030064.112 23504516

[B22] Bitsika V. Sharpley C. F. Andronicos N. M. Agnew L. L. (2015). Hypothalamus-pituitary-adrenal axis daily fluctuation, anxiety and age interact to predict cortisol concentrations in boys with an autism spectrum disorder. Physiol. Behav. 138, 200–207. 10.1016/j.physbeh.2014.11.010 25446203

[B23] Boeckx C. (2011). The emergence of the language faculty, from a biolinguistic point of view, in Oxford Handbook of Language Evolution, eds Tallerman M. Gibson K. (Oxford: Oxford University Press), 492–501.

[B24] Boeckx C. Benítez-Burraco A. (2014a). The shape of the human language-ready brain. Front. Psychol. 5:282. 10.3389/fpsyg.2014.00282 24772099 PMC3983487

[B25] Boeckx C. Benítez-Burraco A. (2014b). Globularity and language-readiness: generating new predictions by expanding the set of genes of interest. Front. Psychol. 5:1324. 10.3389/fpsyg.2014.01324 25505436 PMC4243498

[B26] Bourgeron T. (2007). The possible interplay of synaptic and clock genes in autism spectrum disorders. Cold Spring Harb. Symp. Quant. Biol. 72, 645–654. 10.1101/sqb.2007.72.020 18419324

[B27] Bourguignon N. Nadig A. Valois D. (2012). The biolinguistics of autism: emergent perspectives. Biolinguistics 6, 124–165.

[B28] Brennaman L. H. Kochlamazashvili G. Stoenica L. Nonneman R. J. Moy S. S. Schachner M.. (2011). Transgenic mice overexpressing the extracellular domain of NCAM are impaired in working memory and cortical plasticity. Neurobiol. Dis. 43, 372–378. 10.1016/j.nbd.2011.04.008 21515372 PMC3129860

[B29] Brincat S. L. Miller E. K. (2015). Frequency-specific hippocampal-prefrontal interactions during associative learning. Nat. Neurosci. 18, 576–581. 10.1038/nn.3954 25706471 PMC4444366

[B30] Brugmann S. A. Allen N. C. James A. W. Mekonnen Z. Madan E. Helms J. A. (2010). A primary cilia-dependent etiology for midline facial disorders. Hum. Mol. Genet. 19, 1577–1592. 10.1093/hmg/ddq030 20106874 PMC2846163

[B31] Burke L. M. Kalpakjian C. Z. Smith Y. R. Quint E. H. (2009). Gynecologic issues of adolescents with Down syndrome, autism, and cerebral palsy. J. Pediatr. Adolesc. Gynecol. 23, 11–15. 10.1016/j.jpag.2009.04.005 19643648

[B32] Buzsáki G. Logothetis N. Singer W. (2013). Scaling brain size, keeping timing: evolutionary preservation of brain rhythms. Neuron 80, 751–764. 10.1016/j.neuron.2013.10.002 24183025 PMC4009705

[B33] Buzsáki G. Watson B. O. (2012). Brain rhythms and neural syntax: implications for efficient coding of cognitive content and neuropsychiatric disease. Dialogues Clin. Neurosci. 14, 345–367. 23393413 10.31887/DCNS.2012.14.4/gbuzsaki PMC3553572

[B34] Caballero I. M. Manuel M. N. Molinek M. Quintana-Urzainqui I. Mi D. Shimogori T.. (2014). Cell-autonomous repression of Shh by transcription factor Pax6 regulates diencephalic patterning by controlling the central diencephalic organizer. Cell Rep. 8, 1405–1418. 10.1016/j.celrep.2014.07.051 25176648 PMC4241467

[B35] Cáceres M. Lachuer J. Zapala M. A. Redmond J. C. Kudo L. Geschwind D. H.. (2003). Elevated gene expression levels distinguish human from non-human primate brains. Proc. Natl. Acad. Sci. U.S.A. 100, 13030–13035. 10.1073/pnas.2135499100 14557539 PMC240739

[B36] Campbell D. B. Sutcliffe J. S. Ebert P. J. Militerni R. Bravaccio C. Trillo S.. (2006). A genetic variant that disrupts MET transcription is associated with autism. Proc. Natl. Acad. Sci. U.S.A. 103, 16834–16839. 10.1073/pnas.0605296103 17053076 PMC1838551

[B37] Campbell D. J. Chang J. Chawarska K. (2014). Early generalized overgrowth in autism spectrum disorder: prevalence rates, gender effects, and clinical outcomes. J. Am. Acad. Child Adolesc. Psychiatry 53, 1063–1073. 10.1016/j.jaac.2014.07.008 25245350 PMC4173120

[B38] Campbell K. Flavin N. Ivens A. Robert B. Buckingham M. Williamson R. (1989). The human homeobox gene HOX7 maps to 4p16.1 and is deleted in Wolf-Hirschhorn syndrome patients. Am. J. Hum. Genet. 45, A179. 10.1007/BF00195823 1969845

[B39] Cao F. Yin A. Wen G. Sheikh A. M. Tauqeer Z. Malik M.. (2012). Alteration of astrocytes and Wnt/β-catenin signaling in the frontal cortex of autistic subjects. J. Neuroinflammation 9:223. 10.1186/1742-2094-9-223 22999633 PMC3544729

[B40] Carruthers P. (2006). The Architecture of Mind. Oxford: Oxford University Press.

[B41] Castermans D. Wilquet V. Parthoens E. Huysmans C. Steyaert J. Swinnen L.. (2003). The neurobeachin gene is disrupted by a translocation in a patient with idiopathic autism. J. Med. Genet. 40, 352–356. 10.1136/jmg.40.5.352 12746398 PMC1735479

[B42] Chawarska K. Campbell D. Chen L. Shic F. Klin A. Chang J. (2011). Early generalized overgrowth in boys with autism. Arch. Gen. Psychiatry 68, 1021–1031. 10.1001/archgenpsychiatry.2011.106 21969460 PMC4878118

[B43] Cheng H. Jiang W. Phillips F. M. Haydon R. C. Peng Y. Zhou L.. (2003). Osteogenic activity of the fourteen types of human bone morphogenetic proteins (BMPs). J. Bone Joint Surg. Am. 85A, 1544–1552. 12925636 10.2106/00004623-200308000-00017

[B44] Cheung J. Petek E. Nakabayashi K. Tsui L. C. Vincent J. B. Scherer S. W. (2001). Identification of the human cortactin-binding protein-2 gene from the autism candidate region at 7q31. Genomics 78, 7–11. 10.1006/geno.2001.6651 11707066

[B45] Chiba S. Lee Y. M. Zhou W. Freed C. R. (2008). Noggin enhances dopamine neuron production from human embryonic stem cells and improves behavioral outcome after transplantation into Parkinsonian rats. Stem Cells 26, 2810–2820. 10.1634/stemcells.2008-0085 18772316

[B46] Cho K. K. Hoch R. Lee A. T. Patel T. Rubenstein J. L. Sohal V. S. (2015). Gamma rhythms link prefrontal interneuron dysfunction with cognitive inflexibility in Dlx5/6(+/−) mice. Neuron 85, 1332–1343. 10.1016/j.neuron.2015.02.019 25754826 PMC4503262

[B47] Cobos I. Long J. E. Thwin M. T. Rubenstein J. L. (2006). Cellular patterns of transcription factor expression in developing cortical interneurons. Cereb. Cortex 16, i82–i88. 10.1093/cercor/bhk003 16766712

[B48] Comings D. E. Wu S. Chiu C. Muhleman D. Sverd J. (1996). Studies of the c-Harvey-Ras gene in psychiatric disorders. Psychiatry Res. 63, 25–32. 10.1016/0165-1781(96)02829-6 8832771

[B49] Correia A. C. Costa M. Moraes F. Bom J. Nóvoa A. Mallo M. (2007). Bmp2 is required for migration but not for induction of neural crest cells in the mouse. Dev. Dyn. 236, 2493–2501. 10.1002/dvdy.21256 17676634

[B50] Courchesne E. Campbell K. Solso S. (2011). Brain growth across the life span in autism: age-specific changes in anatomical pathology. Brain Res. 1380, 138–145. 10.1016/j.brainres.2010.09.101 20920490 PMC4500507

[B51] Courchesne E. Pierce K. (2005). Why the frontal cortex in autism might be talking only to itself: local over-connectivity but long-distance disconnection. Curr. Opin. Neurobiol. 15, 225–230. 10.1016/j.conb.2005.03.001 15831407

[B52] Dauger S. Guimiot F. Renolleau S. Levacher B. Boda B. Mas C.. (2001). MASH-1/RET pathway involvement in development of brain stem control of respiratory frequency in newborn mice. Physiol. Genomics 7, 149–157. 10.1152/physiolgenomics.00056.2001 11773601

[B53] Deacon T. W. (2009). Relaxed selection and the role of epigenesis in the evolution of language, in Oxford Handbook of Developmental Behavioral Neuroscience, eds Blumberg M. Freeman J. Robinson J. (New York, NY: Oxford University Press), 730–752.

[B54] Delbroek H. Steyaert J. Legius E. (2011). An 8.9 year old girl with autism and Gorlin syndrome. Eur J. Paediatr. Neurol. 15, 268–270. 10.1016/j.ejpn.2010.12.001 21190878

[B55] Devanna P. Middelbeek J. Vernes S. C. (2014). FOXP2 drives neuronal differentiation by interacting with retinoic acid signaling pathways. Front. Cell. Neurosci. 8:305. 10.3389/fncel.2014.00305 25309332 PMC4176457

[B56] di Rocco F. Baujat G. Arnaud E. Rénier D. Laplanche J. L. Daire V. C.. (2014). Clinical spectrum and outcomes in families with coronal synostosis and TCF12 mutations. Eur. J. Hum. Genet. 22, 1413–1416. 10.1038/ejhg.2014.57 24736737 PMC4231413

[B57] Doi M. Cho S. Yujnovsky I. Hirayama J. Cermakian N. Cato A. C.. (2007). Light-inducible and clock-controlled expression of MAP kinase phosphatase 1 in mouse central pacemaker neurons. J. Biol. Rhythms 22, 127–139. 10.1177/0748730406298332 17440214

[B58] Drerup C. M. Wiora H. M. Topczewski J. Morris J. A. (2009). Disc1 regulates foxd3 and sox10 expression, affecting neural crest migration and differentiation. Development 136, 2623–2632. 10.1242/dev.030577 19570850 PMC2709068

[B59] Dutt S. Kléber M. Matasci M. Sommer L. Zimmermann D. R. (2006). Versican V0 and V1 guide migratory neural crest cells. J. Biol. Chem. 17, 12123–12131. 10.1074/jbc.M510834200 16510447

[B60] Edery P. Lyonnet S. Mulligan L. M. Pelet A. Dow E. Abel L.. (1994). Mutations of the RET proto-oncogene in Hirschsprung's disease. Nature 367, 378–380. 10.1038/367378a0 8114939

[B61] Eigsti I. M. Bennetto L. Dadlani M. (2007). Beyond pragmatics: morphosyntactic development in autism. J. Autism Dev. Disord. 37, 1007–1023. 10.1007/s10803-006-0239-2 17089196

[B62] El-Baz F. Hamza R. T. Ayad M. S. Mahmoud N. H. (2014). Hyperandrogenemia in male autistic children and adolescents: relation to disease severity. Int. J. Adolesc. Med. Health 26, 79–84. 10.1515/ijamh-2012-0116 23612632

[B63] Esghaei M. Daliri M. R. Treue S. (2015). Attention decreases phase-amplitude coupling, enhancing stimulus discriminability in cortical area MT. Front. Neural Circuits 9:82. 10.3389/fncir.2015.00082 26733820 PMC4686998

[B64] Etchamendy N. Enderlin V. Marighetto A. Pallet V. Higueret P. Jaffard R. (2003). Vitamin A deficiency and relational memory deficit in adult mice: relationships with changes in brain retinoid signalling. Behav. Brain Res. 145, 37–49. 10.1016/S0166-4328(03)00099-8 14529804

[B65] Eyles D. W. (2010). Vitamin D and autism: does skin colour modify risk? Acta Paediatr. 99, 645–647. 10.1111/j.1651-2227.2010.01797.x 20219042

[B66] Farmer C. Butter E. Mazurek M. O. Cowan C. Lainhart J. Cook E. H.. (2015). Aggression in children with autism spectrum disorders and a clinic-referred comparison group. Autism 19, 281–291. 10.1177/1362361313518995 24497627 PMC4331245

[B67] Fedorenko E. Thompson-Schill S. L. (2014). Re-working the language network. Trends Cogn. Sci. 18, 120–126. 10.1016/j.tics.2013.12.006 24440115 PMC4091770

[B68] Fernell E. Olsson V. A. Karlgren-Leitner C. Norlin B. Hagberg B. Gillberg C. (1999). Autistic disorders in children with CHARGE association. Dev. Med. Child Neurol. 41, 270–272. 10.1017/S0012162299000572 10355813

[B69] Filová B. Ostatníková D. Celec P. Hodosy J. (2013). The effect of testosterone on the formation of brain structures. Cells Tissues Organs 197, 169–177. 10.1159/000345567 23306974

[B70] Fisher S. E. Francks C. Marlow A. J. MacPhie I. L. Newbury D. F. Cardon L. R.. (2002). Independent genome-wide scans identify a chromosome 18 quantitative-trait locus influencing dyslexia. Nat. Genet. 30, 86–91. 10.1038/ng792 11743577

[B71] Fletcher P. T. Whitaker R. T. Tao R. DuBray M. B. Froehlich A. Ravichandran C.. (2010). Microstructural connectivity of the arcuate fasciculus in adolescents with high-functioning autism. Neuroimage 51, 1117–1125. 10.1016/j.neuroimage.2010.01.083 20132894 PMC2966943

[B72] Franchini L. F. Pollard K. S. (2015). Genomic approaches to studying human-specific developmental traits. Development 142, 3100–3112. 10.1242/dev.120048 26395139

[B73] Fuchs S. Herzog D. Sumara G. Büchmann-Møller S. Civenni G. Wu X.. (2009). Stage-specific control of neural crest stem cell proliferation by the small rho GTPases Cdc42 and Rac1. Cell Stem Cell 4, 236–247. 10.1016/j.stem.2009.01.017 19265663

[B74] Fukumoto A. Hashimoto T. Ito H. Nishimura M. Tsuda Y. Miyazaki M.. (2008). Growth of head circumference in autistic infants during the first year of life. J. Autism Dev. Disord. 38, 411–418. 10.1007/s10803-007-0405-1 17647099

[B75] Gajavelli S. Wood P. M. Pennica D. Whittemore S. R. Tsoulfas P. (2004). BMP signaling initiates a neural crest differentiation program in embryonic rat CNS stem cells. Exp. Neurol. 188, 205–223. 10.1016/j.expneurol.2004.03.026 15246821

[B76] Gao Y. Meng D. Sun N. Zhu Z. Zhao R. Lu C.. (2014). Clock upregulates intercellular adhesion molecule-1 expression and promotes mononuclear cells adhesion to endothelial cells. Biochem. Biophys. Res. Commun. 443, 586–591. 10.1016/j.bbrc.2013.12.022 24333415

[B77] Geschwind G. H. State M. W. (2015). Gene hunting in autism spectrum disorder: on the path to precision medicine. Lancet Neurol. 14, 1109–1120. 10.1016/S1474-4422(15)00044-7 25891009 PMC4694565

[B78] Ghahramani Seno M. M. Hu P. Gwadry F. G. Pinto D. Marshall C. R. Casallo G.. (2011). Gene and miRNA expression profiles in autism spectrum disorders. Brain Res. 1380, 85–97. 10.1016/j.brainres.2010.09.046 20868653

[B79] Ghanem N. Yu M. Poitras L. Rubenstein J. L. Ekker M. (2008). Characterization of a distinct subpopulation of striatal projection neurons expressing the Dlx genes in the basal ganglia through the activity of the I56ii enhancer. Dev. Biol. 322, 415–424. 10.1016/j.ydbio.2008.07.029 18706405

[B80] Ginsberg M. R. Rubin R. A. Falcone T. Ting A. H. Natowicz M. R. (2012). Brain transcriptional and epigenetic associations with autism. PLoS ONE 7:e44736. 10.1371/journal.pone.0044736 22984548 PMC3440365

[B81] Gitton Y. Benouaiche L. Vincent C. Heude E. Soulika M. Bouhali K.. (2011). Dlx5 and Dlx6 expression in the anterior neural fold is essential for patterning the dorsal nasal capsule. Development 138, 897–903. 10.1242/dev.057505 21270050

[B82] Glatt S. J. Cohen O. S. Faraone S. V. Tsuang M. T. (2011). Dysfunctional gene splicing as a potential contributor to neuropsychiatric disorders. Am. J. Med. Genet. B Neuropsychiatr. Genet. 156, 382–392. 10.1002/ajmg.b.31181 21438146 PMC3082621

[B83] Glejzer A. Laudet E. Leprince P. Hennuy B. Poulet C. Shakhova O.. (2011). Wnt1 and BMP2: two factors recruiting multipotent neural crest progenitors isolated from adult bone marrow. Cell. Mol. Life Sci. 68, 2101–2114. 10.1007/s00018-010-0558-5 20976520 PMC11114799

[B84] Gokhman D. Lavi E. Prüfer K. Fraga M. F. Riancho J. A. Kelso J.. (2014). Reconstructing the DNA methylation maps of the Neandertal and the Denisovan. Science 344, 523–527. 10.1126/science.1250368 24786081

[B85] Gómez-Lado C. Eirís-Puñal J. Blanco-Barca O. del Río-Latorre E. Fernández-Redondo V. Castro-Gago M. (2004). Hypomelanosis of Ito. a possibly under-diagnosed heterogeneous neurocutaneous syndrome. Rev. Neurol. 38, 223–228. 14963848

[B86] Gordon C. T. Tan T. Y. Benko S. Fitzpatrick D. Lyonnet S. Farlie P. G. (2009). Long-range regulation at the SOX9 locus in development and disease. J. Med. Genet. 46, 649–656. 10.1136/jmg.2009.068361 19473998

[B87] Hahamy A. Behrmann M. Malach R. (2015). The idiosyncratic brain: distortion of spontaneous connectivity patterns in autism spectrum disorder. Nat. Rev. Neurosci. 18, 302–309. 10.1038/nn.3919 25599222

[B88] Hale C. L. Niederriter A. N. Green G. E. Martin D. M. (2016). Atypical phenotypes associated with pathogenic CHD7 variants and a proposal for broadening CHARGE syndrome clinical diagnostic criteria. Am. J. Med. Genet. A. 170, 344–354. 10.1002/ajmg.a.37435 26590800 PMC5102387

[B89] Hall M. H. Taylor G. Sham P. Schulze K. Rijsdijk F. Picchioni M.. (2011). The early auditory gamma-band response is heritable and a putative endophenotype of schizophrenia. Schizophr. Bull. 37, 778–787. 10.1093/schbul/sbp134 19946013 PMC3122286

[B90] Hamilton A. Marshal M. P. Murray P. J. (2011). Autism spectrum disorders and menstruation. J. Adolesc. Health 49, 443–445. 10.1016/j.jadohealth.2011.01.015 21939879

[B91] Hamza R. T. Hewedi D. H. Ismail M. A. (2010). Basal and adrenocorticotropic hormone stimulated plasma cortisol levels among Egyptian autistic children: relation to disease severity. Ital. J. Pediatr. 36, 71. 10.1186/1824-7288-36-71 21034507 PMC2987909

[B92] Hansen S. M. Berezin V. Bock E. (2008). Signaling mechanisms of neurite outgrowth induced by the cell adhesion molecules NCAM and N-cadherin. Cell Mol. Life Sci. 65, 3809–3821. 10.1007/s00018-008-8290-0 18791849 PMC11131707

[B93] Hare B. Tomasello M. (2005). Human-like social skills in dogs? Trends Cogn. Sci. 9, 439–444. 10.1016/j.tics.2005.07.003 16061417

[B94] Hari L. Miescher I. Shakhova O. Suter U. Chin L. Taketo M.. (2012). Temporal control of neural crest lineage generation by Wnt/β-catenin signaling. Development 139, 2107–2117. 10.1242/dev.073064 22573620

[B95] Hattori T. Shimizu S. Koyama Y. Emoto H. Matsumoto Y. Kumamoto N.. (2014). DISC1 (disrupted-in-schizophrenia-1) regulates differentiation of oligodendrocytes. PLoS ONE 9:e88506. 10.1371/journal.pone.0088506 24516667 PMC3917910

[B96] Hauser M. (2009). The possibility of impossible cultures. Nature 460, 190–196. 10.1038/460190a 19587759

[B97] Hauth I. de Bruijn Y. G. Staal W. Buitelaar J. K. Rommelse N. N. (2014). Testing the extreme male brain theory of autism spectrum disorder in a familial design. Autism Res. 7, 491–500. 10.1002/aur.1384 24777834

[B98] Heffner H. E. Heffner R. S. (1986). Effect of unilateral and bilateral auditory cortex lesions on the discrimination of vocalizations by Japanese macaques. J. Neurophysiol. 56, 683–701. 3783215 10.1152/jn.1986.56.3.683

[B99] Hergüner A. Hergüner S. (2016). Association between age at menarche and autistic traits in Turkish university students. Am. J. Hum. Biol. 28, 44–47. 10.1002/ajhb.22739 25994409

[B100] Hill A. P. Zuckerman K. E. Hagen A. D. Kriz D. J. Duvall S. W. van Santen J.. (2014). Aggressive behavior problems in children with autism spectrum disorders: prevalence and correlates in a large clinical sample. Res. Autism Spectr. Disord. 8, 1121–1133. 10.1016/j.rasd.2014.05.006 25221619 PMC4160737

[B101] Hill D. S. Cabrera R. Wallis Schultz D. Zhu H. Lu W. Finnell R. H.. (2015). Autism-like behavior and epigenetic changes associated with autism as consequences of *in utero* exposure to environmental pollutants in a mouse model. Behav. Neurol. 2015:426263. 10.1155/2015/426263 26586927 PMC4637446

[B102] Hollier L. P. Mattes E. Maybery M. T. Keelan J. A. Hickey M. Whitehouse A. J. (2013). The association between perinatal testosterone concentration and early vocabulary development: a prospective cohort study. Biol. Psychol. 92, 212–215. 10.1016/j.biopsycho.2012.10.016 23153707

[B103] Hollocks M. J. Howlin P. Papadopoulos A. S. Khondoker M. Simonoff E. (2014). Differences in HPA-axis and heart rate responsiveness to psychosocial stress in children with autism spectrum disorders with and without co-morbid anxiety. Psychoneuroendocrinology 46, 32–45. 10.1016/j.psyneuen.2014.04.004 24882156

[B104] Holt R. Sykes N. H. Conceição I. C. Cazier J. B. Anney R. J. Oliveira G.. (2012). CNVs leading to fusion transcripts in individuals with autism spectrum disorder. Eur. J. Hum. Genet. 20, 1141–1147. 10.1038/ejhg.2012.73 22549408 PMC3476715

[B105] Hu V. W. Sarachana T. Sherrard R. M. Kocher K. M. (2015). Investigation of sex differences in the expression of RORA and its transcriptional targets in the brain as a potential contributor to the sex bias in autism. Mol. Autism 6:7. 10.1186/2040-2392-6-7 26056561 PMC4459681

[B106] Huang Y. T. Iwamoto K. Kurosaki T. Nasu M. Ueda S. (2005). The neuronal POU transcription factor Brn-2 interacts with Jab1, a gene involved in the onset of neurodegenerative diseases. Neurosci. Lett. 382, 175–178. 10.1016/j.neulet.2005.03.008 15911144

[B107] Ingudomnukul E. Baron-Cohen S. Wheelwright S. Knickmeyer R. (2007). Elevated rates of testosterone-related disorders in women with autism spectrum conditions. Horm. Behav. 51, 597–604. 10.1016/j.yhbeh.2007.02.001 17462645

[B108] Ishii M. Arias A. C. Liu L. Chen Y. B. Bronner M. E. Maxson R. E. (2012). A stable cranial neural crest cell line from mouse. Stem Cells Dev. 21, 3069–3080. 10.1089/scd.2012.0155 22889333 PMC3495126

[B109] Jacobson L. (2014). Hypothalamic-pituitary-adrenocortical axis: neuropsychiatric aspects. Compr. Physiol. 4, 715–738. 10.1002/cphy.c130036 24715565

[B110] Jeste S. S. Geschwind D. H. (2014). Disentangling the heterogeneity of autism spectrum disorder through genetic findings. Nat. Rev. Neurol. 10, 74–81. 10.1038/nrneurol.2013.278 24468882 PMC4125617

[B111] Jia F. Wang B. Shan L. Xu Z. Staal W. G. Du L. (2015). Core symptoms of autism improved after vitamin D supplementation. Pediatrics 135, e196–e198. 10.1542/peds.2014-2121 25511123

[B112] Jia L. Cheng L. Raper J. (2005). Slit/Robo signaling is necessary to confine early neural crest cells to the ventral migratory pathway in the trunk. Dev. Biol. 282, 411–421. 10.1016/j.ydbio.2005.03.021 15950606

[B113] Jiang W. Yu Q. Gong M. Chen L. Wen E. Y. Bi Y.. (2012). Vitamin A deficiency impairs postnatal cognitive function via inhibition of neuronal calcium excitability in hippocampus. J. Neurochem. 121, 932–943. 10.1111/j.1471-4159.2012.07697.x 22352986

[B114] Jiang Y. H. Yuen R. K. Jin X. Wang M. Chen N. Wu X.. (2013). Detection of clinically relevant genetic variants in autism spectrum disorder by whole-genome sequencing. Am. J. Hum. Genet. 93, 249–263. 10.1016/j.ajhg.2013.06.012 23849776 PMC3738824

[B115] Jones D. L. Howard M. A. Stanco A. Rubenstein J. L. Baraban S. C. (2011). Deletion of Dlx1 results in reduced glutamatergic input to hippocampal interneurons. J. Neurophysiol. 105, 1984–1991. 10.1152/jn.00056.2011 21325686 PMC3094166

[B116] Jones E. G. Rubenstein J. L. R. (2004). Expression of regulatory genes during differentiation of thalamic nuclei in mouse and monkey. J. Comp. Neurol. 477, 55–80. 10.1002/cne.20234 15281080

[B117] Justice C. M. Yagnik G. Kim Y. Peter I. Jabs E. W. Erazo M.. (2012). A genome-wide association study identifies susceptibility loci for nonsyndromic sagittal craniosynostosis near BMP2 and within BBS9. Nat. Genet. 44, 1360–1364. 10.1038/ng.2463 23160099 PMC3736322

[B118] Kanduri C. Kantojärvi K. Salo P. M. Vanhala R. Buck G. Blancher C.. (2016). The landscape of copy number variations in Finnish families with autism spectrum disorders. Autism Res. 9, 9–16. 10.1002/aur.1502 26052927

[B119] Kasamatsu S. Hachiya A. Higuchi K. Ohuchi A. Kitahara T. Boissy R. E. (2008). Production of the soluble form of KIT, s-KIT, abolishes stem cell factor-induced melanogenesis in human melanocytes. J. Invest. Dermatol. 128, 1763–1772. 10.1038/jid.2008.9 18239612

[B120] Katafuchi T. Li A. J. Hirota S. Kitamura Y. Hori T. (2000). Impairment of spatial learning and hippocampal synaptic potentiation in c-kit mutant rats. Learn. Mem. 7, 383–392. 10.1101/lm.33900 11112797 PMC311355

[B121] Kaufmann W. E. Cortell R. Kau A. S. Bukelis I. Tierney E. Gray R. M.. (2004). Autism spectrum disorder in fragile X syndrome: communication, social interaction, and specific behaviors. Am. J. Med. Genet. A. 129, 225–234. 10.1002/ajmg.a.30229 15326621

[B122] Khadka D. Luo T. Sargent T. D. (2006). Msx1 and Msx2 have shared essential functions in neural crest but may be dispensable in epidermis and axis formation in Xenopus. Int. J. Dev. Biol. 50, 499–502. 10.1387/ijdb.052115dk 16586351

[B123] Kilsby A. J. Cruwys M. Kukendrajah C. Russell-Eggitt I. Raglan E. Rajput K.. (2013). Homozygosity for piebaldism with a proven KIT mutation resulting in depigmentation of the skin and hair, deafness, developmental delay and autism spectrum disorder. Clin. Dysmorphol. 22, 64–67. 10.1097/MCD.0b013e32835e8ce5 23399981

[B124] Kim K. C. Lee D. K. Go H. S. Kim P. Choi C. S. Kim J. W.. (2014). Pax6-dependent cortical glutamatergic neuronal differentiation regulates autism-like behavior in prenatally valproic acid-exposed rat offspring. Mol. Neurobiol. 49, 512–528. 10.1007/s12035-013-8535-2 24030726

[B125] Kokovay E. Wang Y. Kusek G. Wurster R. Lederman P. Lowry N.. (2012). VCAM1 is essential to maintain the structure of the SVZ niche and acts as an environmental sensor to regulate SVZ lineage progression. Cell Stem Cell. 11, 220–230. 10.1016/j.stem.2012.06.016 22862947

[B126] Konopka G. Bomar J. M. Winden K. Coppola G. Jonsson Z. O. Gao F.. (2009). Human-specific transcriptional regulation of CNS development genes by FOXP2. Nature 462, 213–217. 10.1038/nature08549 19907493 PMC2778075

[B127] Kopell N. J. Gritton H. J. Whittington M. A. Kramer M. A. (2014). Beyond the connectome: the dynome. Neuron 83, 1319–1328. 10.1016/j.neuron.2014.08.016 25233314 PMC4169213

[B128] Kraus P. Lufkin T. (2006). Dlx homeobox gene control of mammalian limb and craniofacial development. Am. J. Med. Genet. A. 140, 1366–1374. 10.1002/ajmg.a.31252 16688724

[B129] Kruszka P. Li D. Harr M. H. Wilson N. R. Swarr D. McCormick E. M.. (2015). Mutations in SPECC1L, encoding sperm antigen with calponin homology and coiled-coil domains 1-like, are found in some cases of autosomal dominant Opitz G/BBB syndrome. J. Med. Genet. 52, 104–110. 10.1136/jmedgenet-2014-102677 25412741 PMC4393015

[B130] Kuhlwilm M. Davierwala A. Pääbo S. (2013). Identification of putative target genes of the transcription factor RUNX2. PLoS ONE 8:e83218. 10.1371/journal.pone.0083218 24349465 PMC3861491

[B131] Kumari D. Usdin K. (2001). Interaction of the transcription factors USF1, USF2, and alpha -Pal/Nrf-1 with the FMR1 promoter. Implications for Fragile X mental retardation syndrome. J. Biol. Chem. 276, 4357–4364. 11058604 10.1074/jbc.M009629200

[B132] Lalani S. R. Safiullah A. M. Fernbach S. D. Harutyunyan K. G. Thaller C. Peterson L.. (2006). Spectrum of CHD7 mutations in 110 individuals with CHARGE syndrome and genotype-phenotype correlation. Am. J. Hum. Genet. 78, 303–314. 10.1086/500273 16400610 PMC1380237

[B135] Lattanzi W. (forthcoming). Molecular genetics of craniosynostosis, in eLS (Chichester: John Wiley & Sons Ltd.). Available online at: http://www.els.net.

[B133] Lattanzi W. Barba M. Novegno F. Massimi L. Tesori V. Tamburrini G. (2013). Lim mineralization protein is involved in the premature calvarial ossification in sporadic craniosynostoses. Bone 52, 474–484. 10.1016/j.bone.2012.09.004 22982077

[B134] Lattanzi W. Bukvic N. Barba M. Tamburrini G. Bernardini C. Michetti F.. (2012). Genetic basis of single-suture synostoses: genes, chromosomes and clinical implications. Childs Nerv. Syst. 28, 1301–1310. 10.1007/s00381-012-1781-1 22872241

[B136] Le Q. V. Isbell L. A. Matsumoto J. Le V. A. Nishimaru H. Hori E. (2016). Snakes elicit earlier, and monkey faces, later, gamma oscillations in macaque pulvinar neurons. Sci. Rep. 6:20595. 10.1038/srep20595 26854087 PMC4744932

[B137] Lee Y. H. Saint-Jeannet J. P. (2011). Sox9 function in craniofacial development and disease. Genesis 49, 200–208. 10.1002/dvg.20717 21309066 PMC3079054

[B138] Lewis J. D. Theilmann R. J. Fonov V. Bellec P. Lincoln A. Evans A. C.. (2013). Callosal fiber length and interhemispheric connectivity in adults with autism: brain overgrowth and underconnectivity. Hum. Brain Mapp. 34, 1685–1695. 10.1002/hbm.22018 22359385 PMC6870247

[B139] Li C. Ito H. Fujita K. Shiwaku H. Qi Y. Tagawa K.. (2013). Sox2 transcriptionally regulates PQBP1, an intellectual disability-microcephaly causative gene, in neural stem progenitor cells. PLoS ONE 8:e68627. 10.1371/journal.pone.0068627 23874697 PMC3713010

[B140] Li J. Feng J. Liu Y. Ho T. V. Grimes W. Ho H. A.. (2015b). BMP-SHH signaling network controls epithelial stem cell fate via regulation of its niche in the developing tooth. Dev. Cell. 33, 125–135. 10.1016/j.devcel.2015.02.021 25865348 PMC4406846

[B141] Li S. Quarto N. Senarath-Yapa K. Grey N. Bai X. Longaker M. T. (2015a). Enhanced activation of canonical Wnt signaling confers mesoderm-derived parietal bone with similar osteogenic and skeletal healing capacity to neural crest-derived frontal bone. PLoS ONE 10:e0138059. 10.1371/journal.pone.0138059 26431534 PMC4592195

[B142] Lin M. Pedrosa E. Shah A. Hrabovsky A. Maqbool S. Zheng D.. (2011). RNA-Seq of human neurons derived from iPS cells reveals candidate long non-coding RNAs involved in neurogenesis and neuropsychiatric disorders. PLoS ONE 6:e23356. 10.1371/journal.pone.0023356 21915259 PMC3168439

[B143] Lindgren K. Folstein S. Tomblin J. B. Tager-Flusberg H. (2009). Language and reading abilities of children with autism spectrum disorders and specific language impairment and their first-degree relatives. Autism Res. 2, 22–38. 10.1002/aur.63 19358305 PMC2806306

[B144] Linkenkaer-Hansen K. Smit D. J. Barkil A. van Beijsterveldt T. E. Brussaard A. B. Boomsma D. I.. (2007). Genetic contributions to long-range temporal correlations in ongoing oscillations. J. Neurosci. 27, 13882–13889. 10.1523/JNEUROSCI.3083-07.2007 18077700 PMC6673639

[B145] Liu H. Ding T. Zhan Y. Feng H. (2015). A novel AXIN2 missense mutation is associated with non-syndromic oligodontia. PLoS ONE 10:e0138221. 10.1371/journal.pone.0138221 26406231 PMC4583461

[B146] Liu X. Somel M. Tang L. Yan Z. Jiang X. Guo S.. (2012). Extension of cortical synaptic development distinguishes humans from chimpanzees and macaques. Genome Res. 22, 611–622. 10.1101/gr.127324.111 22300767 PMC3317144

[B147] Liu Y. Jin Y. Li J. Seto E. Kuo E. Yu W.. (2013). Inactivation of Cdc42 in neural crest cells causes craniofacial and cardiovascular morphogenesis defects. Dev. Biol. 383, 239–252. 10.1016/j.ydbio.2013.09.013 24056078

[B148] Lopes Floro K. Artap S. T. Preis J. I. Fatkin D. Chapman G.. (2011). Loss of Cited2 causes congenital heart disease by perturbing left-right patterning of the body axis. Hum. Mol. Genet. 20, 1097–1110. 10.1093/hmg/ddq554 21224256

[B149] López-Bendito G. Flames N. Ma L. Fouquet C. Di Meglio T. Chedotal A.. (2007). Robo1 and Robo2 cooperate to control the guidance of major axonal tracts in the mammalian forebrain. J. Neurosci. 27, 3395–3407. 10.1523/JNEUROSCI.4605-06.2007 17392456 PMC6672128

[B150] Luo T. Wagner E. Dräger U. C. (2009). Integrating retinoic acid signaling with brain function. Dev. Psychol. 45, 139–150. 10.1037/0012-1649.45.1.139 19209997

[B151] Luo X. Ding L. Xu J. Chegini N. (2005). Gene expression profiling of leiomyoma and myometrial smooth muscle cells in response to transforming growth factor-beta. Endocrinology 146, 1097–1118. 10.1210/en.2004-1377 15604209

[B152] Luppanapornlarp S. Leelataweewud P. Putongkam P. Ketanont S. (2010). Periodontal status and orthodontic treatment need of autistic children. World J. Orthod. 11, 256–261. 20877735

[B153] Lupyan G. Dale R. (2010). Language structure is partly determined by social structure. PLoS ONE 5:e8559. 10.1371/journal.pone.0008559 20098492 PMC2798932

[B154] Maekawa M. Iwayama Y. Nakamura K. Sato M. Toyota T. Ohnishi T.. (2009). A novel missense mutation (Leu46Val) of PAX6 found in an autistic patient. Neurosci. Lett. 462, 267–271. 10.1016/j.neulet.2009.07.021 19607881

[B155] Malenfant P. Liu X. Hudson M. L. Qiao Y. Hrynchak M. Riendeau N.. (2012). Association of GTF2i in the Williams-Beuren syndrome critical region with autism spectrum disorders. J. Autism Dev. Disord. 42, 1459–1469. 10.1007/s10803-011-1389-4 22048961

[B156] Maliepaard M. Mathijssen I. M. Oosterlaan J. Okkerse J. M. (2014). Intellectual, behavioral, and emotional functioning in children with syndromic craniosynostosis. Pediatrics 133, e1608–e1615. 10.1542/peds.2013-3077 24864183

[B157] Mallo M. (2001). Formation of the middle ear: recent progress on the developmental and molecular mechanisms. Dev. Biol. 231, 410–419. 10.1006/dbio.2001.0154 11237469

[B158] Manouilenko I. Eriksson J. M. Humble M. B. Bejerot S. (2014). Minor physical anomalies in adults with autism spectrum disorder and healthy controls. Autism Res. Treat. 2014:743482. 10.1155/2014/743482 24782925 PMC3982266

[B159] Mansour S. Offiah A. C. McDowall S. Sim P. Tolmie J. Hall C. (2002). The phenotype of survivors of campomelic dysplasia. J. Med. Genet. 39, 597–602. 10.1136/jmg.39.8.597 12161603 PMC1735206

[B160] Marcos-Mondéjar P. Peregrín S. Li J. Y. Carlsson L. Tole S. López-Bendito G. (2012). The lhx2 transcription factor controls thalamocortical axonal guidance by specific regulation of robo1 and robo2 receptors. J. Neurosci. 32, 4372–4385. 10.1523/JNEUROSCI.5851-11.2012 22457488 PMC6622047

[B161] Maricic T. Günther V. Georgiev O. Gehre S. Curlin M. Schreiweis C.. (2013). A recent evolutionary change affects a regulatory element in the human FOXP2 gene. Mol. Biol. Evol. 30, 844–852. 10.1093/molbev/mss271 23197593

[B162] Martin P. M. Yang X. Robin N. Lam E. Rabinowitz J. S. Erdman C. A. (2013). A rare WNT1 missense variant overrepresented in ASD leads to increased Wnt signal pathway activation. Transl. Psychiatry 3:e301. 10.1038/tp.2013.75 24002087 PMC3784764

[B163] Maruyama T. Jeong J. Sheu T. J. Hsu W. (2016). Stem cells of the suture mesenchyme in craniofacial bone development, repair and regeneration. Nat. Commun. 7:10526. 10.1038/ncomms10526 26830436 PMC4740445

[B164] Matsuo T. Osumi-Yamashita N. Noji S. Ohuchi H. Koyama E. Myokai F.. (1993). A mutation in the Pax-6 gene in rat small eye is associated with impaired migration of midbrain. Nat. Genet. 3, 299–304. 10.1038/ng0493-299 7981749

[B165] McKinsey G. Lindtner S. Trzcinski B. Visel A. Pennacchio L. A. Huylebroeck D.. (2013). Dlx1and2-dependent expression of Zfhx1b (Sip1, Zeb2) regulates the fate switch between cortical and striatal interneurons. Neuron 77, 83–98. 10.1016/j.neuron.2012.11.035 23312518 PMC3547499

[B166] McLarren K. W. Litsiou A. Streit A. (2003). DLX5 positions the neural crest and preplacode region at the border of the neural plate. Dev. Biol. 259, 34–47. 10.1016/S0012-1606(03)00177-5 12812786

[B167] Mercier S. Dubourg C. Garcelon N. Campillo-Gimenez B. Gicquel I. Belleguic M.. (2011). New findings for phenotype-genotype correlations in a large European series of holoprosencephaly cases. J. Med. Genet. 48, 752–760. 10.1136/jmedgenet-2011-100339 21940735 PMC3386902

[B168] Metzis V. Courtney A. D. Kerr M. C. Ferguson C. Rondón Galeano M. C. Parton R. G.. (2013). Patched1 is required in neural crest cells for the prevention of orofacial clefts. Hum. Mol. Genet. 22, 5026–5035. 10.1093/hmg/ddt353 23900075

[B169] Meyer M. Kircher M. Gansauge M. T. Li H. Racimo F. Mallick S.. (2012). A high-coverage genome sequence from an archaic Denisovan individual. Science 338, 222–226. 10.1126/science.1224344 22936568 PMC3617501

[B170] Mikita N. Hollocks M. J. Papadopoulos A. S. Aslani A. Harrison S. Leibenluft E.. (2015). Irritability in boys with autism spectrum disorders: an investigation of physiological reactivity. J. Child Psychol. Psychiatry 56, 1118–1126. 10.1111/jcpp.12382 25626926 PMC4737220

[B171] Milet C. Maczkowiak F. Roche D. D. Monsoro-Burq A. H. (2013). Pax3 and Zic1 drive induction and differentiation of multipotent, migratory, and functional neural crest in Xenopus embryos. Proc. Natl. Acad. U.S.A. 110, 5528–5533. 10.1073/pnas.1219124110 PMC3619367 23509273

[B172] Miller J. A. Ding S. L. Sunkin S. M. Smith K. A. Ng L. Szafer A.. (2014). Transcriptional landscape of the prenatal human brain. Nature 508, 199–206. 10.1038/nature13185 24695229 PMC4105188

[B173] Ming J. E. Kaupas M. E. Roessler E. Brunner H. G. Golabi M. Tekin M.. (2002). Mutations in PATCHED-1, the receptor for SONIC HEDGEHOG, are associated with holoprosencephaly. Hum. Genet. 110, 297–301. 10.1007/s00439-002-0695-5 11941477

[B174] Mithen S. (1996). The Prehistory of the Mind. London: Thames and Hudson.

[B175] Miyano M. Horike S. Cai S. Oshimura M. Kohwi-Shigematsu T. (2008). DLX5 expression is monoallelic and Dlx5 is up-regulated in the Mecp2-null frontal cortex. J. Cell Mol. Med. 12, 1188–1191. 10.1111/j.1582-4934.2008.00377.x 18537997 PMC3597349

[B176] Moreno-Ramos O. A. Olivares A. M. Haider N. B. de Autismo L. C. Lattig M. C. (2015). Whole-exome sequencing in a South American cohort links ALDH1A3, FOXN1 and retinoic acid regulation pathways to autism spectrum disorders. PLoS ONE 10:e0135927. 10.1371/journal.pone.0135927 26352270 PMC4564166

[B177] Morey-Canellas J. Sivagamasundari U. Barton H. (2003). A case of autism in a child with Apert's syndrome. Eur. Child Adolesc. Psychiatry 12, 100–102. 10.1007/s00787-003-0289-3 12664274

[B178] Mosconi M. W. Cody-Hazlett H. Poe M. D. Gerig G. Gimpel-Smith R. Piven J. (2009). Longitudinal study of amygdala volume and joint attention in 2- to 4-year-old children with autism. Arch. Gen. Psychiatry 66, 509–516. 10.1001/archgenpsychiatry.2009.19 19414710 PMC3156446

[B179] Murphy C. M. Deeley Q. Daly E. M. Ecker C. O'Brien F. M. Hallahan B.. (2012). Anatomy and aging of the amygdala and hippocampus in autism spectrum disorder: an *in vivo* magnetic resonance imaging study of Asperger syndrome. Autism Res. 5, 3–12. 10.1002/aur.227 21948742

[B180] Murphy E. (2015a). The brain dynamics of linguistic computation. Front. Psychol. 6:1515. 10.3389/fpsyg.2015.01515 26528201 PMC4602109

[B181] Murphy E. (2015b). Labels, cognomes and cyclic computation: an ethological perspective. Front. Psychol. 6:715. 10.3389/fpsyg.2015.00715 26089809 PMC4453271

[B182] Murphy E. (2016a). The human oscillome and its explanatory potential. Biolinguistics 10, 6–20.

[B183] Murphy E. (2016b). Evolutionary monkey oscillomics: generating linking hypotheses from preserved brain rhythms. Theor. Linguist. 42, 117–137. 10.1515/tl-2016-0005

[B184] Murphy E. Benítez-Burraco A. (in press). Language deficits in schizophrenia autism as related oscillatory connectomopathies: an evolutionary account. Neurosci. Biobehav. Rev. 10.1101/044198 27475632

[B185] Nakashima N. Yamagata T. Mori M. Kuwajima M. Suwa K. Momoi M. Y. (2010). Expression analysis and mutation detection of DLX5 and DLX6 in autism. Brain Dev. 32, 98–104. 10.1016/j.braindev.2008.12.021 19195802

[B186] Naqvi S. Cole T. Graham J. M. Jr. (2000). Cole-Hughes macrocephaly syndrome and associated autistic manifestations. Am. J. Med. Genet. 94, 149–152. 10982971 10.1002/1096-8628(20000911)94:2<149::aid-ajmg7>3.0.co;2-#

[B187] Nelson C. S. Fuller C. K. Fordyce P. M. Greninger A. L. Li H. DeRisi J. L. (2013). Microfluidic affinity and ChIP-seq analyses converge on a conserved FOXP2-binding motif in chimp and human, which enables the detection of evolutionarily novel targets. Nucleic Acids Res. 41, 5991–6004. 10.1093/nar/gkt259 23625967 PMC3695516

[B188] Nguyen A. Rauch T. A. Pfeifer G. P. Hu V. W. (2010). Global methylation profiling of lymphoblastoid cell lines reveals epigenetic contributions to autism spectrum disorders and a novel autism candidate gene, *RORA*, whose protein product is reduced in autistic brain. FASEB. J. 24, 3036–3051. 10.1096/fj.10-154484 20375269 PMC2909294

[B189] Nordahl C. W. Lange N. Li D. D. Barnett L. A. Lee A. Buonocore M. H.. (2011). Brain enlargement is associated with regression in preschool-age boys with autism spectrum disorders. Proc. Natl. Acad. Sci. U.S.A. 108, 20195–20200. 10.1073/pnas.1107560108 22123952 PMC3250128

[B190] Obafemi-Ajayi T. Miles J. H. Takahashi T. N. Qi W. Aldridge K. Zhang M.. (2015). Facial structure analysis separates autism spectrum disorders into meaningful clinical subgroups. J. Autism Dev. Disord. 45, 1302–1317. 10.1007/s10803-014-2290-8 25351828

[B191] Obaydi H. Puri B. K. (2008). Prevalence of premenstrual syndrome in autism: a prospective observer-rated study. J. Int. Med. Res. 36, 268–272. 10.1177/147323000803600208 18380936

[B192] Oosterveen T. Kurdija S. Alekseenko Z. Uhde C. W. Bergsland M. Sandberg M.. (2012). Mechanistic differences in the transcriptional interpretation of local and long-range Shh morphogen signaling. Dev. Cell 23, 1006–1019. 10.1016/j.devcel.2012.09.015 23153497

[B193] Oosterveen T. Kurdija S. Ensterö M. Uhde C. W. Bergsland M. Sandberg M.. (2013). SoxB1-driven transcriptional network underlies neural-specific interpretation of morphogen signals. Proc. Natl. Acad. Sci. U.S.A. 110, 7330–7335. 10.1073/pnas.1220010110 23589857 PMC3645538

[B194] Ortiz-Mantilla S. Choe M. S. Flax J. Grant P. E. Benasich A. A. (2010). Associations between the size of the amygdala in infancy and language abilities during the preschool years in normally developing children. Neuroimage 49, 2791–2799. 10.1016/j.neuroimage.2009.10.029 19850137

[B195] Pääbo S. (2014). The human condition-a molecular approach. Cell 157, 216–226. 10.1016/j.cell.2013.12.036 24679537

[B196] Paratore C. Brugnoli G. Lee H. Y. Suter U. Sommer L. (2002). The role of the Ets domain transcription factor Erm in modulating differentiation of neural crest stem cells. Dev. Biol. 250, 168–180. 10.1006/dbio.2002.0795 12297104

[B197] Paumard-Hernández B. Berges-Soria J. Barroso E. Rivera-Pedroza C. I. Pérez-Carrizosa V. Benito-Sanz S.. (2015). Expanding the mutation spectrum in 182 Spanish probands with craniosynostosis: identification and characterization of novel TCF12 variants. Eur. J. Hum. Genet. 23, 907–914. 10.1038/ejhg.2014.205 25271085 PMC4463497

[B198] Peterson K. A. Nishi Y. Ma W. Vedenko A. Shokri L. Zhang X.. (2012). Neural-specific Sox2 input and differential Gli-binding affinity provide context and positional information in Shh-directed neural patterning. Genes Dev. 26, 2802–2816. 10.1101/gad.207142.112 23249739 PMC3533082

[B199] Piard J. Rozé V. Czorny A. Lenoir M. Valduga M. Fenwick A. L.. (2015). TCF12 microdeletion in a 72-year-old woman with intellectual disability. Am. J. Med. Genet. A. 167A, 1897–1901. 10.1002/ajmg.a.37083 25871887 PMC4654244

[B200] Pisano S. Milone A. Gemo I. Masi G. (2014). High-functioning autism spectrum disorder associated with CHARGE syndrome: a case report. Clin. Dysmorphol. 23, 35–37. 10.1097/MCD.0000000000000014 24172694

[B201] Plante E. Boliek C. Binkiewicz A. Erly W. K. (1996). Elevated androgen, brain development and language/learning disabilities in children with congenital adrenal hyperplasia. Dev. Med. Child Neurol. 38, 423–437. 10.1111/j.1469-8749.1996.tb15100.x 8698150

[B202] Platt M. L. Seyfarth R. M. Cheney D. L. (2016). Adaptations for social cognition in the primate brain. Philos. Trans. R. Soc. Lond. B Biol. Sci. 371:20150096. 10.1098/rstb.2015.0096 26729935 PMC4745018

[B203] Plouhinec J. L. Roche D. D. Pegoraro C. Figueiredo A. L. Maczkowiak F. Brunet L. J.. (2014). Pax3 and Zic1 trigger the early neural crest gene regulatory network by the direct activation of multiple key neural crest specifiers. Dev. Biol. 386, 461–472. 10.1016/j.ydbio.2013.12.010 24360906 PMC3962137

[B204] Poeppel D. (2012). The maps problem and the mapping problem: two challenges for a cognitive neuroscience of speech and language. Cogn. Neuropsychol. 29, 34–55. 10.1080/02643294.2012.710600 23017085 PMC3498052

[B205] Poitras L. Yu M. Lesage-Pelletier C. Macdonald R. B. Gagné J. P. Hatch G.. (2010). An SNP in an ultraconserved regulatory element affects Dlx5/Dlx6 regulation in the forebrain. Development 137, 3089–3097. 10.1242/dev.051052 20702565 PMC3188590

[B206] Preis J. I. Wise N. Solloway M. J. Harvey R. P. Sparrow D. B. Dunwoodie S. L. (2006). Generation of conditional Cited2 null alleles. Genesis 44, 579–583. 10.1002/dvg.20251 17133411

[B207] Preissler M. A. (2008). Associative learning of pictures and words by low-functioning children with autism. Autism 12, 231–248. 10.1177/1362361307088753 18445733

[B208] Prüfer K. Racimo F. Patterson N. Jay F. Sankararaman S. Sawyer S.. (2014). The complete genome sequence of a Neanderthal from the Altai Mountains. Nature 505, 43–49. 10.1038/nature12886 24352235 PMC4031459

[B209] Ramirez-Villegas J. F. Logothetis N. K. Besserve M. (2015). Sharp wave-ripple complexes in a reduced model of the hippocampal CA3-CA1 network of the macaque monkey. BMC Neurosci. 16(Suppl. 1):P15. 10.1186/1471-2202-16-S1-P15

[B210] Raznahan A. Wallace G. L. Antezana L. Greenstein D. Lenroot R. Thurm A.. (2013). Compared to what? Early brain overgrowth in autism and the perils of population norms. Biol. Psychiatry 74, 563–575. 10.1016/j.biopsych.2013.03.022 23706681 PMC4837958

[B211] Read A. P. Newton V. E. (1997). Waardenburg syndrome. J. Med. Genet. 34, 656–665. 10.1136/jmg.34.8.656 9279758 PMC1051028

[B212] Reser J. E. (2014). Solitary mammals provide an animal model for autism spectrum disorders. J. Comp. Psychol. 128, 99–113. 10.1037/a0034519 24188618

[B213] Rice D. P. Connor E. C. Veltmaat J. M. Lana-Elola E. Veistinen L. Tanimoto Y.. (2010). Gli3Xt-J/Xt-J mice exhibit lambdoid suture craniosynostosis which results from altered osteoprogenitor proliferation and differentiation. Hum. Mol. Genet. 19, 3457–3467. 10.1093/hmg/ddq258 20570969 PMC2916710

[B214] Rogers J. Gibbs R. A. (2014). Comparative primate genomics: emerging patterns of genome content and dynamics. Nat. Rev. Genet. 15, 347–359. 10.1038/nrg3707 24709753 PMC4113315

[B215] Rønn L. C. Berezin V. Bock E. (2000). The neural cell adhesion molecule in synaptic plasticity and ageing. Int. J. Dev. Neurosci. 18, 193–199. 10.1016/S0736-5748(99)00088-X 10715574

[B216] Rothschild G. Sottas C. M. Kissel H. Agosti V. Manova K. Hardy M. P.. (2003). A role for Kit receptor signaling in Leydig cell steroidogenesis. Biol. Reprod. 69, 925–932. 10.1095/biolreprod.102.014548 12773427

[B217] Ruest L. B. Hammer R. E. Yanagisawa M. Clouthier D. E. (2003). Dlx5/6-enhancer directed expression of Cre recombinase in the pharyngeal arches and brain. Genesis 37, 188–194. 10.1002/gene.10247 14666512 PMC2830754

[B218] Saadi I. Alkuraya F. S. Gisselbrecht S. S. Goessling W. Cavallesco R. Turbe-Doan A.. (2011). Deficiency of the cytoskeletal protein SPECC1L leads to oblique facial clefting. Am. J. Hum. Genet. 89, 44–55. 10.1016/j.ajhg.2011.05.023 21703590 PMC3135813

[B219] Sacco R. Gabriele S. Persico A. M. (2015). Head circumference and brain size in autism spectrum disorder: a systematic review and meta-analysis. Psychiatry Res. 234, 239–251. 10.1016/j.pscychresns.2015.08.016 26456415

[B220] Sage S. R. Jürgens U. Ehret G. (2006). Audio-vocal interaction in the pontine brainstem during self-initiated vocalization in the squirrel monkey. Eur. J. Neurosci. 23, 3297–3308. 10.1111/j.1460-9568.2006.04835.x 16820019

[B221] Sajan S. A. Rubenstein J. L. Warchol M. E. Lovett M. (2011). Identification of direct downstream targets of Dlx5 during early inner ear development. Hum. Mol. Genet. 20, 1262–1273. 10.1093/hmg/ddq567 21227998 PMC3049351

[B222] Sarachana T. Hu V. W. (2013a). Genome-wide identification of transcriptional targets of RORA reveals direct regulation of multiple genes associated with autism spectrum disorder. Mol. Autism. 4:14. 10.1186/2040-2392-4-14 23697635 PMC3665583

[B223] Sarachana T. Hu V. W. (2013b). Differential recruitment of coregulators to the RORA promoter adds another layer of complexity to gene (dys) regulation by sex hormones in autism. Mol. Autism 4:39. 10.1186/2040-2392-4-39 24119295 PMC4016566

[B224] Sarachana T. Xu M. Wu R. C. Hu V. W. (2011). Sex hormones in autism: androgens and estrogens differentially and reciprocally regulate RORA, a novel candidate gene for autism. PLoS ONE 6:e17116. 10.1371/journal.pone.0017116 21359227 PMC3040206

[B225] Schipul S. E. Keller T. A. Just M. A. (2011). Inter-regional brain communication and its disturbance in autism. Front. Syst. Neurosci. 5:10. 10.3389/fnsys.2011.00010 21390284 PMC3046360

[B226] Schumann C. M. Barnes C. C. Lord C. Courchesne E. (2009). Amygdala enlargement in toddlers with autism related to severity of social and communication impairments. Biol. Psychiatry 66, 942–949. 10.1016/j.biopsych.2009.07.007 19726029 PMC2795360

[B227] Scott-Van Zeeland A. A. Abrahams B. S. Alvarez-Retuerto A. I. Sonnenblick L. I. Rudie J. D. Ghahremani D.. (2010b). Altered functional connectivity in frontal lobe circuits is associated with variation in the autism risk gene CNTNAP2. Sci. Transl. Med. 2:56ra80. 10.1126/scitranslmed.3001344 21048216 PMC3065863

[B228] Scott-Van Zeeland A. A. McNealy K. Wang A. T. Sigman M. Bookheimer S. Y. Dapretto M. (2010a). No neural evidence of statistical learning during exposure to artificial languages in children with autism spectrum disorders. Biol. Psychiatry 68, 345–351. 10.1016/j.biopsych.2010.01.011 20303070 PMC3229830

[B229] Seed A. Tomasello M. (2010). Primate cognition. Top Cogn. Sci. 2, 407–419. 10.1111/j.1756-8765.2010.01099.x 25163869

[B230] Segklia A. Seuntjens E. Elkouris M. Tsalavos S. Stappers E. Mitsiadis T. A.. (2012). Bmp7 regulates the survival, proliferation, and neurogenic properties of neural progenitor cells during corticogenesis in the mouse. PLoS ONE 7:e34088. 10.1371/journal.pone.0034088 22461901 PMC3312908

[B231] Sharma V. P. Fenwick A. L. Brockop M. S. McGowan S. J. Goos J. A. Hoogeboom A. J.. (2013). Mutations in TCF12, encoding a basic helix-loop-helix partner of TWIST1, are a frequent cause of coronal craniosynostosis. Nat. Genet. 45, 304–307. 10.1038/ng.2531 23354436 PMC3647333

[B232] Smit D. J. Smith A. G. Parsons P. G. Muscat G. E. Sturm R. A. (2000). Domains of Brn2 that mediate homodimerization and interaction with general and melanocytic transcription factors. Eur. J. Biochem. 267, 6413–6422. 10.1046/j.1432-1327.2000.01737.x 11029584

[B233] Smith L. E. Barker E. T. Seltzer M. M. Abbeduto L. Greenberg J. S. (2012). Behavioral phenotype of fragile X syndrome in adolescence and adulthood. Am. J. Intellect. Dev. Disabil. 117, 1–17. 10.1352/1944-7558-117.1.1 22264109 PMC3388941

[B234] Somel M. Liu X. Tang L. Yan Z. Hu H. Guo S.. (2011). MicroRNA-driven developmental remodeling in the brain distinguishes humans from other primates. PLoS Biol. 9:e1001214. 10.1371/journal.pbio.1001214 22162950 PMC3232219

[B235] Spelke E. (2003). What makes us smart? in Language in Mind, eds Gentner D. Goldin-Meadow S. (Cambridge, MA: MIT Press), 277–311.

[B236] Sperber S. M. Saxena V. Hatch G. Ekker M. (2008). Zebrafish dlx2a contributes to hindbrain neural crest survival, is necessary for differentiation of sensory ganglia and functions with dlx1a in maturation of the arch cartilage elements. Dev. Biol. 314, 59–70. 10.1016/j.ydbio.2007.11.005 18158147

[B237] Spratt E. G. Nicholas J. S. Brady K. T. Carpenter L. A. Hatcher C. R. Meekins K. A.. (2012). Enhanced cortisol response to stress in children in autism. J. Autism Dev. Disord. 42, 75–81. 10.1007/s10803-011-1214-0 21424864 PMC3245359

[B238] Stefanatos G. A. Baron I. S. (2011). The ontogenesis of language impairment in autism: a neuropsychological perspective. Neuropsychol. Rev. 21, 252–270. 10.1007/s11065-011-9178-6 21842186

[B239] Steiger J. L. Bandyopadhyay S. Farb D. H. Russek S. J. (2004). cAMP response element-binding protein, activating transcription factor-4, and upstream stimulatory factor differentially control hippocampal GABABR1a and GABABR1b subunit gene expression through alternative promoters. J. Neurosci. 24, 6115–6126. 10.1523/JNEUROSCI.1200-04.2004 15240803 PMC6729677

[B240] Stein C. M. Schick J. H. Gerry Taylor H. Shriberg L. D. Millard C. Kundtz-Kluge A.. (2004). Pleiotropic effects of a chromosome 3 locus on speech-sound disorder and reading. Am. J. Hum. Genet. 74, 283–297. 10.1086/381562 14740317 PMC1181926

[B241] Sterelny K. (2011). From hominins to humans: how sapiens became behaviourally modern. Philos. Trans. R. Soc. Lond. B Biol. Sci. 366, 809–822. 10.1098/rstb.2010.0301 21320896 PMC3048993

[B242] St Pourcain B. Cents R. A. Whitehouse A. J. Haworth C. M. Davis O. S. O'Reilly P. F.. (2014). Common variation near ROBO2 is associated with expressive vocabulary in infancy. Nat. Commun. 5, 4831. 10.1038/ncomms5831 25226531 PMC4175587

[B243] Suda S. Iwata K. Shimmura C. Kameno Y. Anitha A. Thanseem I.. (2011). Decreased expression of axon-guidance receptors in the anterior cingulate cortex in autism. Mol. Autism. 2:14. 10.1186/2040-2392-2-14 21859478 PMC3177773

[B244] Szklarczyk D. Franceschini A. Wyder S. Forslund K. Heller D. Huerta-Cepas J.. (2015). STRING v10: protein-protein interaction networks, integrated over the tree of life. Nucleic Acids Res. 43, D447–D452. 10.1093/nar/gku1003 25352553 PMC4383874

[B245] Tabuchi A. Sakaya H. Kisukeda T. Fushiki H. Tsuda M. (2002). Involvement of an upstream stimulatory factor as well as cAMP-responsive element-binding protein in the activation of brain-derived neurotrophic factor gene promoter I. J. Biol. Chem. 277, 35920–35931. 10.1074/jbc.M204784200 12114522

[B246] Tager-Flusberg H. (2006). Defining language phenotypes in autism. Clin. Neurosci. Res. 6, 219–224. 10.1016/j.cnr.2006.06.007

[B247] Tager-Flusberg H. Paul R. Lord C. (2005). Language and communication in autism, in Handbook of Autism and Pervasive Developmental Disorders, eds Volkmar F. R. Paul R. Klin A. Cohen D. (New York, NY: Wiley), 335–364.

[B248] Taylor J. L. Corbett B. A. (2014). A review of rhythm and responsiveness of cortisol in individuals with autism spectrum disorders. Psychoneuroendocrinology 49, 207–228. 10.1016/j.psyneuen.2014.07.015 25108163 PMC4165710

[B249] Thomas J. (2014). Self-Domestication and Language Evolution. Ph.D. thesis. University of Edinburgh, Edinburgh.

[B250] Tomarken A. J. Han G. T. Corbett B. A. (2015). Temporal patterns, heterogeneity, and stability of diurnal cortisol rhythms in children with autism spectrum disorder. Psychoneuroendocrinology 62, 217–226. 10.1016/j.psyneuen.2015.08.016 26318632 PMC4945957

[B251] Toro R. Konyukh M. Delorme R. Leblond C. Chaste P. Fauchereau F.. (2010). Key role for gene dosage and synaptic homeostasis in autism spectrum disorders. Trends Genet. 26, 363–372. 10.1016/j.tig.2010.05.007 20609491

[B252] Tripi G. Roux S. Canziani T. Bonnet Brilhault F. Barthélémy C. Canziani F. (2008). Minor physical anomalies in children with autism spectrum disorder. Early Hum. Dev. 84, 217–223. 10.1016/j.earlhumdev.2007.04.005 17566672

[B253] Twigg S. R. Forecki J. Goos J. A. Richardson I. C. Hoogeboom A. J. van den Ouweland A. M.. (2015). Gain-of-function mutations in ZIC1 are associated with coronal craniosynostosis and learning disability. Am. J. Hum. Genet. 97, 378–388. 10.1016/j.ajhg.2015.07.007 26340333 PMC4564895

[B254] Twigg S. R. Vorgia E. McGowan S. J. Peraki I. Fenwick A. L. Sharma V. P.. (2013). Reduced dosage of ERF causes complex craniosynostosis in humans and mice and links ERK1/2 signaling to regulation of osteogenesis. Nat. Genet. 45, 308–313. 10.1038/ng.2539 23354439 PMC3683605

[B255] Twigg S. R. Wilkie A. O. (2015). New insights into craniofacial malformations. Hum. Mol. Genet. 24, R50–R59. 10.1093/hmg/ddv228 26085576 PMC4571997

[B256] Tyas D. A. Pearson H. Rashbass P. Price D. J. (2003). Pax6 regulates cell adhesion during cortical development. Cereb. Cortex 13, 612–619. 10.1093/cercor/13.6.612 12764036

[B257] Uchino J. Suzuki M. Hoshino K. Nomura Y. Segawa M. (2001). Development of language in Rett syndrome. Brain Dev. 23, S233–S235. 10.1016/S0387-7604(01)00367-9 11738880

[B258] Uddin L. Q. Supekar K. Menon V. (2013). Reconceptualizing functional brain connectivity in autism from a developmental perspective. Front. Hum. Neurosci. 7:458. 10.3389/fnhum.2013.00458 23966925 PMC3735986

[B259] Uittenbogaard M. Chiaramello A. (2002). Expression of the bHLH transcription factor Tcf12 (ME1) gene is linked to the expansion of precursor cell populations during neurogenesis. Brain Res. Gene Expr. Patterns 1, 115–121. 10.1016/S1567-133X(01)00022-9 15018808 PMC2757747

[B260] Valverde F. García C. López-Mascaraque L. De Carlos J. A. (2000). Development of the mammillothalamic tract in normal and Pax-6 mutant mice. J. Comp. Neurol. 419, 485–504. 10.1002/(SICI)1096-9861(20000417)419:4<485::AID-CNE6>3.0.CO;2-1 10742717

[B261] van Daalen E. Swinkels S. H. Dietz C. van Engeland H. Buitelaar J. K. (2007). Body length and head growth in the first year of life in autism. Pediatr. Neurol. 37, 324–330. 10.1016/j.pediatrneurol.2007.06.006 17950417

[B262] Veenstra-VanderWeele J. Cook E. H. (2004). Molecular genetics of autism spectrum disorder. Mol. Psychiatry 9, 819–832. 10.1038/sj.mp.4001505 15197396

[B263] Vernes S. C. Oliver P. L. Spiteri E. Lockstone H. E. Puliyadi R. Taylor J. M.. (2011). Foxp2 regulates gene networks implicated in neurite outgrowth in the developing brain. PLoS Genet. 7:e1002145. 10.1371/journal.pgen.1002145 21765815 PMC3131290

[B264] Vissers L. E. L. M. van Ravenswaaij C. M. A. Admiraal R. Hurst J. A. de Vries B. B. A. Janssen I. M.. (2004). Mutations in a new member of the chromodomain gene family cause CHARGE syndrome. Nat. Genet. 36, 955–957. 10.1038/ng1407 15300250

[B265] Voineagu I. Wang X. Johnston P. Lowe J. K. Tian Y. Horvath S.. (2011). Transcriptomic analysis of autistic brain reveals convergent molecular pathology. Nature 474, 380–384. 10.1038/nature10110 21614001 PMC3607626

[B266] von Aster M. Zachmann M. Brandeis D. Wohlrab G. Richner M. Steinhausen H. C. (1997). Psychiatric, neuropediatric, and neuropsychological symptoms in a case of hypomelanosis of Ito. Eur. Child Adolesc. Psychiatry 6, 227–233. 10.1007/s007870050034 9443002

[B267] Walenski M. Tager-Flusberg H. Ullman M. (2006). Language in autism, in Understanding Autism: From Basic Neuroscience to Treatment, eds Moldin S. O. Rubenstein J. L. R. (Boca Raton, FL: Taylor and Francis Books), 175–203.

[B268] Wang Q. Moore M. J. Adelmant G. Marto J. A. Silver P. A. (2013). PQBP1, a factor linked to intellectual disability, affects alternative splicing associated with neurite outgrowth. Genes. Dev. 27, 615–626. 10.1101/gad.212308.112 23512658 PMC3613609

[B269] Wang Y. Billon C. Walker J. K. Burris T. P. (2016). Therapeutic effect of a synthetic RORα/γ agonist in an animal model of autism. ACS Chem. Neurosci. 7, 143–148. 10.1021/acschemneuro.5b00159 26625251 PMC4759619

[B270] Wang Y. Dye C. A. Sohal V. Long J. E. Estrada R. C. Roztocil T.. (2010). Dlx5 and Dlx6 regulate the development of parvalbumin-expressing cortical interneurons. J. Neurosci. 30, 5334–5345. 10.1523/JNEUROSCI.5963-09.2010 20392955 PMC2919857

[B271] Wray A. Grace G. W. (2007). The consequences of talking to strangers: evolutionary corollaries of socio-cultural influences on linguistic form. Lingua 117, 543–578. 10.1523/JNEUROSCI.5963-09.2010

[B272] Watanabe H. Takahashi K. Isa T. (2015). Phase locking of β oscillation in electrocorticography (ECoG) in the monkey motor cortex at the onset of EMGs and 3D reaching movements, in Engineering in Medicine and Biology Society (EMBC), 37th Annual International Conference of the IEEE (Milan), 55–58. 10.1109/EMBC.2015.7318299 26736199

[B273] Weaver K. N. Watt K. E. Hufnagel R. B. Navajas Acedo J. Linscott L. L. Sund K. L.. (2015). Acrofacial dysostosis, Cincinnati type, a mandibulofacial dysostosis syndrome with limb anomalies, is caused by POLR1A dysfunction. Am. J. Hum. Genet. 96, 765–774. 10.1016/j.ajhg.2015.03.011 25913037 PMC4570288

[B274] Wilkins A. S. Wrangham R. W. Fitch W. T. (2014). The “domestication syndrome” in mammals: a unified explanation based on neural crest cell behavior and genetics. Genetics 197, 795–808. 10.1534/genetics.114.165423 25024034 PMC4096361

[B275] Williams J. M. Beck T. F. Pearson D. M. Proud M. B. Cheung S. W. Scott D. A. (2009). A 1q42 deletion involving DISC1, DISC2, and TSNAX in an autism spectrum disorder. Am. J. Med. Genet. A. 149A, 1758–1762. 10.1002/ajmg.a.32941 19606485 PMC2909829

[B276] Willsey A. J. State M. W. (2015). Autism spectrum disorders: from genes to neurobiology. Curr. Opin. Neurobiol. 30, 92–99. 10.1016/j.conb.2014.10.015 25464374 PMC4586254

[B277] Wilson N. R. Olm-Shipman A. J. Acevedo D. S. Palaniyandi K. Hall E. G. Kosa E.. (2016). SPECC1L deficiency results in increased adherens junction stability and reduced cranial neural crest cell delamination. Sci. Rep. 6:17735. 10.1038/srep17735 26787558 PMC4726231

[B278] Wyatt A. W. Osborne R. J. Stewart H. Ragge N. K. (2010). Bone morphogenetic protein 7 (BMP7) mutations are associated with variable ocular, brain, ear, palate, and skeletal anomalies. Hum. Mutat. 31, 781–787. 10.1002/humu.21280 20506283

[B279] Wynn T. Coolidge F. (2011). How to Think Like a Neanderthal. Oxford: Oxford University Press.

[B280] Yang Z. Matsumoto A. Nakayama K. Jimbo E. F. Kojima K. Nagata K.. (2016). Circadian-relevant genes are highly polymorphic in autism spectrum disorder patients. Brain Dev. 38, 91–99. 10.1016/j.braindev.2015.04.006 25957987

[B281] Yu H. M. Jerchow B. Sheu T. J. Liu B. Costantini F. Puzas J. E.. (2005). The role of Axin2 in calvarial morphogenesis and craniosynostosis. Development 132, 1995–2005. 10.1242/dev.01786 15790973 PMC1828115

[B282] Yuge K. Kataoka A. Yoshida A. C. Itoh D. Aggarwal M. Mori S.. (2011). Region-specific gene expression in early postnatal mouse thalamus. J. Comp. Neurol. 519, 544–561. 10.1002/cne.22532 21192083

[B283] Zhang Y. Sun Y. Wang F. Wang Z. Peng Y. Li R. (2012). Downregulating the canonical Wnt/β-catenin signaling pathway attenuates the susceptibility to autism-like phenotypes by decreasing oxidative stress. Neurochem. Res. 37, 1409–1419. 10.1007/s11064-012-0724-2 22374471

[B284] Zheng F. Wang L. Jia M. Yue W. Ruan Y. Lu T.. (2011). Evidence for association between Disrupted-in-Schizophrenia 1 (DISC1) gene polymorphisms and autism in Chinese Han population: a family-based association study. Behav. Brain Funct. 7:14. 10.1186/1744-9081-7-14 21569632 PMC3113723

[B285] Zhou H. Hu S. Matveev R. Yu Q. Li J. Khaitovich P.. (2015). A chronological atlas of natural selection in the human genome during the past half-million years. BioRxiv. [Epub ahead of print]. 10.1101/018929

